# *Bifidobacterium animalis* subsp. *lactis* Bi-07 supports lactose digestion in vitro and in randomized, placebo- and lactase-controlled clinical trials

**DOI:** 10.1093/ajcn/nqac264

**Published:** 2022-09-23

**Authors:** Pia Rasinkangas, Sofia D Forssten, Maija Marttinen, Alvin Ibarra, Gordana Bothe, Jouni Junnila, Ralf Uebelhack, Yves Donazzolo, Arthur C Ouwehand

**Affiliations:** Health & Biosciences, International Flavors & Fragrances Inc. (IFF), Kantvik, Finland; Health & Biosciences, International Flavors & Fragrances Inc. (IFF), Kantvik, Finland; Health & Biosciences, International Flavors & Fragrances Inc. (IFF), Kantvik, Finland; Health & Biosciences, International Flavors & Fragrances Inc. (IFF), Kantvik, Finland; analyze & realize GmbH, Berlin, Germany; Oy 4Pharma Ltd., Turku, Finland; analyze & realize GmbH, Berlin, Germany; Eurofins Optimed SAS, Gières, France; Health & Biosciences, International Flavors & Fragrances Inc. (IFF), Kantvik, Finland

**Keywords:** probiotic, lactose maldigestion, lactose intolerance, lactase, *Bifidobacterium lactis* Bi-07, in vitro, randomized controlled trial, breath hydrogen concentration

## Abstract

**Background:**

Probiotics may alleviate lactose maldigestion.

**Objectives:**

The objective was to select a probiotic with high lactase activity and compare it with lactase and placebo in clinical trials.

**Methods:**

Bacterial cultures were screened for lactase activity in a model of the upper gastrointestinal (GI) tract. *Bifidobacterium animalis* subsp. *lactis* Bi-07 (Bi-07) counts were adjusted in subsequent experiments to correspond to 4500 Food Chemicals Codex (FCC) units of lactase, the amount in the European Food Safety Authority (EFSA)-approved health claim. Two crossover clinical trials, Booster Alpha and Booster Omega, were performed in participants with lactose intolerance, where 2 × 10^12^ CFUs Bi-07, 4662 FCC lactase, or placebo was consumed simultaneously with a lactose challenge, with 1-wk washouts between challenges. The trial designs were identical except for the source of lactose. Breath hydrogen concentration (BHC) was measured to assess the effect of the investigational products on lactose digestion, for which incremental area under the curve (iAUC) was the primary outcome. Peak BHC, cumulative BHC, and GI symptoms were secondary outcomes.

**Results:**

Bi-07 was superior to placebo in reducing BHC [iAUC, parts per million (ppm) ∙ h] in both trials (Booster Alpha: geometric least square mean ratio: 0.462; 95% CI: 0.249, 0.859; *P =* 0.016; Booster Omega: 0.227; 95% CI: 0.095, 0.543; *P* = 0.001). Lactase was superior to placebo in Booster Alpha (0.190; 95% CI: 0.102, 0.365; *P* < 0.001) but not Booster Omega (0.493; 95% CI: 0.210, 1.156; *P* = 0.102). Noninferiority of Bi-07 compared with lactase was observed in Booster Omega (0.460; 95% CI: 0.193, 1.096; *P* = 0.079; CI upper limit < 1.25 noninferiority margin). Odds of abdominal pain (compared with placebo: 0.32, *P* = 0.036) and flatulence (compared with placebo: 0.25, *P* = 0.007) were lower with lactase in Booster Alpha. Increased odds of nausea were seen with Bi-07 (compared with placebo: 4.0, *P* = 0.005) in Booster Omega.

**Conclusions:**

Bi-07 has high lactase activit*y*, and in 2 clinical trials, it supported lactose digestion in individuals with lactose intolerance.

These trials were registered at clinicaltrials.gov as NCT03659747 (Booster Alpha) and NCT03814668 (Booster Omega).

## Introduction

People with lactose maldigestion express low amounts of β-galactosidase—more commonly called lactase—in the small intestine (1). The resulting inability to digest lactose properly is referred to as lactose intolerance, due to lactase deficiency (2). In primary lactase deficiency, the most common type of lactase deficiency, intestinal lactase production declines over time, usually from age 2 y (2). Lactose maldigestion can lead to lactose malabsorption, in which lactose enters the colon and is fermented by resident bacteria, resulting in symptoms of lactose intolerance, such as abdominal pain, bloating, flatulence, diarrhea, and nausea (2, 3). Factors of these symptoms include the amount of lactose that is ingested, intestinal lactase activity, gastric emptying rate, gastrointestinal (GI) transit time, and gut microbiota composition and activity (1, 4).

A diagnosis of lactose intolerance that is based solely on symptoms is unreliable, owing to overlap with other conditions with similar symptomatologies, including irritable bowel syndrome, celiac disease, and small intestinal bacterial overgrowth (2, 3). Thus, breath hydrogen concentration (BHC) during a lactose challenge is the most convenient and reliable diagnostic tool for assessing lactose maldigestion, based on its objectivity and low invasiveness (5). Recently, it has been suggested that BHC measurements should be combined with a newly published validated symptom questionnaire (6, 7). In addition to BHC measurement, gene and lactose tolerance tests (glucose measurements from the blood during a lactose challenge), which require blood sampling, are used in diagnostics (4).

Certain bifidobacteria can metabolize lactose owing to endogenous lactase production (8); this property could be exploited to aid in lactose digestion if such bacteria are consumed with lactose-containing food or beverage. Bifidobacterial lactase production can be stimulated by culturing these strains in lactose-containing culture medium (9, 10). *Bifidobacterium animalis* subsp. *lactis* Bi-07 (hereafter referred to as Bi-07) is a probiotic that was originally isolated from a healthy human. Bi-07 has been studied extensively, and its safety has been well established in vitro and in vivo (11), with further proof of safe consumption from several decades of commercial use. It improves GI symptoms when used alone or in combination with other probiotics (12–14).

The effects of probiotics on lactose maldigestion have been examined extensively since the clinical recognition of the condition in the 1970s (15), but the results have often been ambiguous owing to poor study design (16). In this study, we addressed this shortcoming by performing a thorough in vitro characterization of the lactase activity of Bi-07 and 2 subsequent clinical trials using modern clinical standards and sufficient statistical power and design to determine its effects on lactose maldigestion.

## Methods

### Bacterial strains

The following bacterial strains and combinations were studied in an in vitro screen using a simulated upper gastrointestinal tract (GIT): Bi-07 ATCC SD5220, *Lactobacillus acidophilus* NCFM ATCC SD5221 (NCFM), *Lactobacillus delbrueckii* subsp. *bulgaricus* Lb-87 ATCC SD6833 (Lb-87), *Streptococcus thermophilus* St-21 ATCC SD5207 (St-21), as well as Bulk Set Y 532, YO-MIX™ 601, and YO-MIX™ 401 starter cultures that contained a selection of lactic acid bacteria. Tests of lactase activity in milk and the clinical studies were performed exclusively with Bi-07. The bacterial strains and strain mixtures were grown in fermentation media that contained milk components. In addition, a form of *L. acidophilus* NCFM that was cultured in the absence of milk components (HOWARU Dophilus) was included to evaluate the effects of the growth medium. Bi-07 and other individual strains were manufactured by Danisco USA, and YO-MIX and Bulk Set Y 532 cultures were manufactured by Danisco Deutschland GmbH.

### Lactase activity in the upper GIT model

Of each bacterium or mixture, 0.1 g freeze-dried powder was suspended in 1 mL water. The number of cells per gram of each strain was as follows: Bi-07, 2.97 × 10^11^; NCFM, 5.73 × 10^10^; HOWARU Dophilus, 1.86 × 10^10^; Lb-87, 1.34 × 10^10^; St-21, 9.86 × 10^9^; Bulk Set Y, 7.61 × 10^10^; YO-MIX 601, 2.16 × 10^10^; and YO-MIX 401, 2.78 × 10^11^. These amounts were determined on a FACS Calibur (BD Biosciences) as described (17).

Lactase activity in the bacterial strains and mixtures was first studied in batch cultures, which comprised pure cultures or defined mixtures of the strains in water. Lactase activity was determined in cell-free extracts, against a standard curve for p-nitrophenyl-β-d-galactopyranoside (N1252, Sigma-Aldrich Finland Oy). Cell-free extracts were prepared by diluting the cultures 1:6 with water, and the resulting suspensions were sonicated 4 times for 15 s each on ice and centrifuged for 5 min at 2000 × *g* at 4°C. Next, 0.3 mL of substrate solution was added to 0.2 mL of supernatant. In the blank sample, 0.3 mL 0.1 M phosphate buffer (pH 7) (30407, Riedel de-Haën/Sigma-Aldrich) was added to 0.2 mL of supernatant. Then, the samples were mixed and incubated for 1 h at 37°C, after which 1.5 mL 0.25 M Na_2_CO_3_ (31432, Riedel de-Haën/Sigma-Aldrich) was added to stop the reaction. The absorbance was measured at 400 nm on a Spectramax 250 (Molecular Devices). The enzymatic activity (nmol · g^−1^ · min^−1^) in the samples was calculated using the following formula: nmol · g^−1^ · min^−1^ = [concentration (ng/mL) × 6 × 2 mL]/[0.2 mL × 139.1 g/mol × 60 min], where 6 is the dilution factor.

The lactase activity of Bi-07, HOWARU Dophilus, and YO-MIX 601 was tested in a simulated upper GIT; 8000 units of laboratory-grade β-galactosidase (cat. no. G5160-125KU, 8 U/mg; Sigma-Aldrich) was used as a positive control. In brief, 0.1 g of each bacterial strain or mixture was suspended in 1 mL phosphate-carbonate buffer (adjusted to pH 6.5) and incubated for 5 min with 1.2 mL α-amylase (A1031, Sigma-Aldrich) at 37°C to simulate the oral cavity. Next, the pH was lowered to 2.5 with HCl (258148, Sigma-Aldrich) to mimic the stomach, and 2.4 mL simulated gastric juice that contained pepsin (P7000, Sigma-Aldrich) was added; the contents were then incubated for 1 h 15 min at 37°C. To simulate the small intestine and duodenum, the pH was neutralized to 6.5 with NaOH (98108.209 VWR), after which 2.4 mL simulated duodenal juice that contained pancreatin and lipase (P3292 and PL3126, Sigma-Aldrich) and 1.2 mL bile juice (B3883, Sigma-Aldrich) were added, and the mixture was incubated for 1 h 30 min at 37°C. Lactase activity was determined as described earlier for samples from the stomach and duodenal stages.

### Lactase activity in milk

Several initial tests were performed to adjust the quantity of Bi-07 to correspond its lactase activity to 4500 Food Chemicals Codex (FCC) units of lactase, a quantity that is commonly used in commercial lactase products and has an EFSA-approved health claim (18) (results not shown). Ultimately, samples that contained 6 g Bi-07, 6 g maltodextrin (Insta Potato Maltodextrin Low Aw, Innovative Food Processors), and 4500 FCC units of lactase from a commercial lactase product (Lactrase, Verman Oy) in 6 g maltodextrin were evaluated for their lactase activity in milk. The samples were mixed into 500 mL fat-free (0 g/100 g), pasteurized, and homogenized milk (Valio Oy) and incubated in closed bottles on a rotating incubator for 6 h at 37°C. Laboratory-grade β-galactosidase (4500 FCC) was used as a control for lactose hydrolysis. Samples were collected during the 6-h incubation at 30-min intervals during the first hour and at 1-h intervals thereafter. The milk was mixed thoroughly before sampling.

To stop the enzymatic activity, the samples were placed in a water bath at 98°C for 10 min. Owing to coagulation of the milk that was treated with Bi-07, the coagulated protein and bacteria were pelleted by centrifugation (4500 × *g*, 10 min, 4°C), and the lactose-containing supernatant was collected. The pH was measured and recorded in the samples.

Further deproteinization and clarification of milk samples were performed per the Carrez clarification kit instructions (cat. no. 1.10537.0001, Millipore, Merck). In brief, 0.5 mL of each sample was diluted in 30 mL distilled water, to which Carrez solution I [containing potassium hexacyanoferrate (II) trihydrate] was added and mixed carefully. Next, 0.5 mL of Carrez solution II (containing zinc sulfate heptahydrate) was added and mixed. The pH of the solution was adjusted to 7.5–8.0 with NaOH to precipitate excess zinc ions as Zn(OH)_2_. The sample solution was then adjusted to 50 mL with distilled water to a final dilution of 1:100. The solution was centrifuged (4500 × *g*, 10 min, 4°C) to separate the insoluble compounds and passed through filter paper (Whatman, Grade 41).

The lactose content in the filtered samples was determined by enzymatic assay (Lactose/d-glucose kit, cat. no. 10 986 119 035, R-Biopharm). In brief, the assay reagents and sample (dilution 1:100) were pipetted into a macrocuvette per the manufacturer's instructions, and the absorbance was measured at 340 nm on a UV spectrophotometer (Ultrospec 1100 pro, Amersham Biosciences). The amount of lactose in the sample was calculated, based on the difference between d-glucose concentrations with and without lactose hydrolysis by lactase in the assay. The experiments were performed in triplicate.

### Clinical trials on the effects of Bi-07 in participants with lactose maldigestion

#### Study principles and ethical approval

Two randomized, double-blind, placebo-controlled and lactase-controlled, crossover, acute lactose challenge clinical trials—termed Booster Alpha and Booster Omega—were performed in accordance with the World Medical Association (WMA) Declaration of Helsinki, the European Medicines Agency (EMA) Guideline on Good Clinical Practice E6 (R2) (CPMP/ICH/135/95), and laws and regulations for clinical research in the involved countries.

Booster Alpha (NCT03659747) was approved by the ethical committee (EC) of Charité, Universitätsmedizin Berlin, Germany (EA1/112/18) and was performed in Berlin by analyze & realize GmbH. The principal investigator (PI) was RU. The active phase of the study began on 11 September, 2018 and ended on 21 December, 2018. The clinical trial protocol was amended once before the start of the study (version 1.1, 25 June, 2018; version approved by the EC).

Booster Omega (NCT03814668) was approved by Comité de Protection des Personnes sud est VI, Clermont-Ferrand, France (CPP Ref. AU 1448, ID-RCB Ref. 2018-A01542-53) and was performed in Gières, Grenoble by Eurofins Optimed SAS, with YD as the PI. The active phase of the study started on 17 October, 2018 and ended on 30 April, 2019. The clinical trial protocol (version 1.0, 9 May, 2018) was amended 3 times: version 1.1, 29 June, 2018 (version submitted to the EC); version 1.2, 25 September, 2018 (approved by the EC); and version 2.0, 12 February, 2019 (sent for information to the EC). In version 2.0 of the protocol, the initial requirement of having each sex comprise ≥40% of the study population was removed owing to difficulties in recruiting suitable participants. Both studies were also approved by the DuPont Human Study Committee before commencement.

All study participants, study site personnel, contract research organization personnel who monitored or conducted the study, representatives of 4Pharma who were involved in the study statistics and data management, and sponsor representatives who designed the study and participated in the study conduct were blinded to individual treatment assignments for the entire study period. Randomization and generation of the randomization lists were performed using the 3 × 3 crossover Williams design (19, 20) per a computerized procedure (SAS System for Windows version 9.3; SAS Institute Inc.) by a randomization expert from 4Pharma, who was not otherwise involved in the study statistics or data management.

Participants were randomly assigned equally to the 6 possible sequences of the 3 treatments: (A) Bi-07, (B) lactase, and (C) placebo, yielding the sequences ABC, ACB, BCA, BAC, CAB, and CBA. The randomization lists that contained details of all participant numbers and study groups were stored as essential documentation in the trial master file only at the end of the study and were unavailable to any person who was actively involved in the studies during the study period. Only in the case of a serious adverse event (SAE) was the list to be made available for pharmacovigilance personnel. The randomization lists were sent directly from 4Pharma to the manufacturer of the sponsor's investigational product (IP), Danisco USA Inc., in Madison, WI, where labeling of the IP was performed. The sponsor's personnel who were involved in the production and labeling of the IP did not participate in the conduct of the studies.

All participants who signed an informed consent form and who entered the formal screening process were assigned a unique screening number automatically through the Viedoc electronic data capture (EDC) system (Viedoc Technologies Ab). On completion of the screen and confirmation that a participant was eligible for participation, the participant was randomly assigned to a treatment by the investigator or a delegate, who assigned a randomization number that was generated by the randomization program in the EDC system. This number corresponded to an IP kit number that was used for a participant for the duration of the study. The site staff then provided the participant with the corresponding IP, mixed in lactose-containing liquid, as described in what follows.

A blind data review (BDR) was performed for each study after the database was locked and before unblinding of the data. Statistical analysis plans (SAPs) and participant classification documents were also signed before the data were unblinded. Statistical and clinical study reports were prepared according to the sponsor's standard operating procedures. The trial master files will be archived in the sponsor's central clinical trial archive facility for ≥25 y. Investigator site files have been archived by the respective clinical sites.

#### Study design

The study design of the 2 clinical trials was identical except regarding the source of lactose: in Booster Alpha the IP was mixed in fat-free milk, whereas an aqueous lactose solution was used in Booster Omega. Milk was used to demonstrate efficacy with a lactose source that is often used in similar studies and the lactose solution was used because it is the recommended source for lactose challenges in the clinical setting (5, 6). The trials included 5 study visits: a prescreening visit (V1); a screening visit (V2); and study visits 1 (V3), 2 (V4), and 3 (V5), during which the IP was administered in random order in a crossover setting.

**FIGURE 1:**
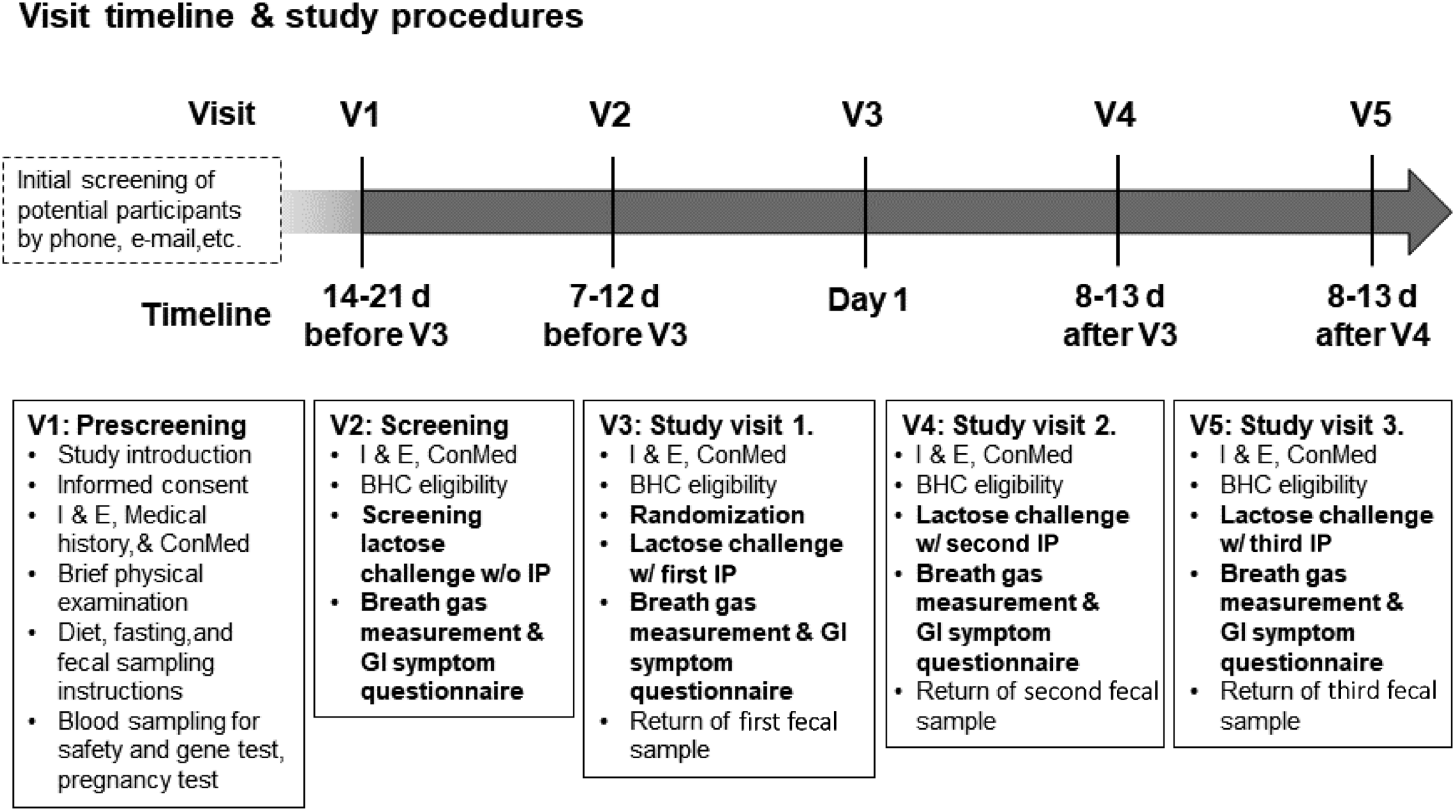
Study design of the clinical trials, Booster Alpha and Booster Omega. BHC, breath hydrogen concentration; BHC eligibility, check-up of possible foods and actions affecting breath gas measurement; ConMed, concomitant medication; GI, gastrointestinal; I & E, inclusion and exclusion criteria review; w/ IP, with Investigational Product; w/o IP, without Investigational Product.

The IPs were *1*) the active product, freeze-dried Bi-07 (2.40 × 10^12^ CFU in Booster Alpha and 2.34 × 10^12^ CFU in Booster Omega); *2*) the active comparator product, lactase (4662 FCC units in both studies, the exact amount was measured from sample IP sachets by Sora Labs per the USP/Dietary Supplements Compendium 2015 standard), with maltodextrin as a carrier; and *3*) placebo (6 g maltodextrin). The quantity of the lactase and placebo IPs was adjusted to 6 g as chosen for Bi-07, to render the IP sachets and their contents indistinguishable from each other in shape and texture. **Figure 1** details the trial design, and the study procedures are explained in what follows.

The sample size calculations (detailed in the “Statistics” section) in the study design phase determined that 34 generally healthy adults were to be randomly assigned to each study. Female and male participants aged 25–60 y (inclusive) with self-declared or medically diagnosed lactose intolerance were invited to participate. In addition, participants were required to have had a positive result on the lactose challenge in the screen at V2—i.e., an increase of >20 parts per million (ppm) in BHC within 3 h from baseline fasting breath hydrogen values (5). **Supplemental Methods 1** provides the complete lists of inclusion and exclusion criteria for each study. The lists were nearly identical, differing in regionally required criteria and specific modifications that were required by the respective investigator or EC.

The assessment of the effects of the IP during the lactose challenge was based on breath hydrogen measurements. The primary objectives of the study were to determine the differences in BHC (ppm) in the lactase and Bi-07 groups compared with placebo and assess the noninferiority of Bi-07 to lactase, based on BHC. Incremental area under curve (iAUC) values for BHC (ppm · h) were calculated to evaluate the primary outcomes (21). The secondary objectives comprised peak breath hydrogen value (C_max_, ppm) and cumulative breath hydrogen (ppm) in the lactase and Bi-07 groups compared with placebo, and noninferiority of Bi-07 compared with lactase was assessed as well. BHC was measured according to the recommendation described in Rezaie et al. (5) and as recommended by the manufacturer of the breath gas analyzer (QuinTron Systems, Inc.).

In addition, information on acute GI symptoms and bowel movements was collected using an experimental (unvalidated) questionnaire that assessed the following: presence and severity of abdominal pain, flatulence, bloating, and nausea on a Likert scale (none, mild, moderate, or severe); presence or absence of vomiting and bowel movements; and stool consistency, based on the Bristol Stool Scale (22, 23). The symptoms on the questionnaire (**Supplemental Methods 2**) were chosen to reflect the most common symptoms in lactose intolerance (24–28).

Baseline fasting BHC, breath methane and carbon dioxide concentrations (ppm), Bi-07 concentrations in feces, and genotyping of known lactase nonpersistence–causing single-nucleotide polymorphisms (SNPs) [-13910*C, -22018*G, -13915*T, -14010*G, and -13907*C (29, 30)] were examined as ancillary analyses. Safety information regarding adverse events (AEs) was collected, and blood safety parameters [hemoglobin, blood lipids (total cholesterol, LDL cholesterol, HDL cholesterol, and TGs), markers of hepatic function (AST, ALT, and γ-glutamyl transferase), markers of kidney function (urea, serum creatinine, sodium, and potassium), and urine pregnancy test (females of child-bearing potential only)] were evaluated during the eligibility evaluation at the screening visit.

#### Details of the study procedures

Breath samples were collected with the AlveoSampler™ Breath Test Kit, and breath gas concentrations were measured using the QuinTron Breath Tracker; all measurements were made per the manufacturer's instructions (QuinTron Systems, Inc.). Breath gases were measured twice at baseline and twice per hour during the 6-h challenge. For each gas, numeric values in ppm were provided by the analyzer.

To prepare for the breath test, participants were asked to brush their teeth in the morning and rinse their mouths with water immediately before the first breath gas measurement at each visit. Participants were also asked to adhere to a restricted diet for 24 h before the breath test and fast for 12 h before the test, as instructed by the manufacturer of the breath gas analyzer (31). Smoking, including exposure to secondhand smoke; chewing gum; and exercise were prohibited in the morning before the breath test. The participants were asked to keep a food diary of the 24-h period before the breath gas measurements to be able to consume the same diet before each breath gas measurement. During the 6-h period, the participants stayed at the site, with the possibility of calm activities such as reading. The participants were allowed to drink water to a maximum of 500 mL, but no food was allowed.

At the screening visit (V2), a lactose challenge was performed without the IP to define the lactose maldigestion status of the participant. Baseline breath gas was measured twice within 15 min before the start of the challenge to obtain a reliable result, after which the participant drank 521 mL fat-free (<0.1 g/100 mL) milk (Molkerei Weihenstephan GmbH & Co. KG; Booster Alpha) or 250 mL aqueous lactose solution (QuinTron Systems, Inc.; Booster Omega), each containing 25 g lactose. The lactose concentration in the milk was confirmed earlier with the Lactose/d-glucose kit (R-Biopharm), as described already. Next, breath gas was measured once immediately (5 min after the start of the challenge) and every 30 min for 6 h after the start of the challenge (for a total of 15 times at each visit). If a participant's BHC values did not rise as required—i.e., by >20 ppm within 3 h during the lactose challenge at the screening—the test could be stopped at 3 h. If willing, the participant could be screened for the study once more at another time point.

At V3–5 (study visits 1–3), the lactose challenge was performed on consumption of the IPs in random order to determine their effect on breath gas values. The study products and lactose-containing liquid were mixed in blenders (Electrolux PerfectMix Model ESB2300, Electrolux), following a procedure on which the study personnel was carefully instructed. The milk and IP mixture was to be consumed within 5 min, whereas the mixture of lactose solution and IP was to be taken within 2.5 min of preparation. A GI symptom questionnaire was completed 15 min before and 5 min after the start of the challenge (after the liquid was consumed and breath gas was measured) and every 60 min for 6 h from the start to the end of the challenge (total of 8 times during each visit). **Supplemental Methods 3** details the fecal sampling and quantification of Bi-07 from feces.

### Statistics

#### In vitro assays

In the quantitative β-galactosidase (lactase) activity assay of the batch cultures, 2 replicate measurements were performed, and 1 was performed for the simulated upper GIT assay; thus, no statistics could be calculated for these experiments.

With regard to the lactase activity in milk, the 3 treatments (maltodextrin used as placebo in the clinical trials, lactase, and Bi-07) were compared by repeated-measures (RM)-ANOVA. The change from the 0-h value was used as the response. This model included treatment, time point (0.5 h, 1 h, 2 h, 3 h, 4 h, 5 h, 6 h), and their interaction as fixed effects and experiment replicate number as a random factor. Least square means (LSMs) were used to estimate differences between the 3 treatments. All statistical calculations were performed by 4Pharma Ltd. using SAS version 9.4 (SAS Institute Inc.).

#### Clinical trials

##### Sample size calculations

Sample sizes were calculated based on previous studies with similar designs, follow-up times, and endpoint definitions (32–34). The decrease in BHC with Bi-07 treatment was assumed to be of a similar extent to that with lactase in these publications. The percentage decreases of mean AUC with lactase in these reports were 17.3% (250 mg dose)/23.7% (500 mg dose) in the DiPalma and Collins (32) study, 63.5% in the Rosado et al. (33) study, and 43.0% in the Sanders et al. (34) study. Only Sanders et al. (34) reported individual AUC data, allowing within-participant differences and their SDs to be estimated. Thus, Sanders et al. (34) was used as the main reference with regard to the sample size calculations.

Based on the reference studies, a 35% decrease in iAUC was selected as the most plausible assumption for the effect size for a probiotic compared with placebo. Based on the data in Sanders et al. (34), the SD for within-participant differences between a probiotic and placebo was assumed to be 0.7 log(AUC). Assuming a 35% decline in iAUC for Bi-07 compared with placebo, an SD of the differences of 0.7 log(AUC) (equaling the square root of the within-participant mean squared error from the crossover ANOVA of 0.495), a 1-sided α-level of 0.025, and an attrition rate of 10%, 30 evaluable participants and 34 randomly assigned participants were needed in total to reach 90% power for the primary hypothesis.

Because the decrease in BHC with Bi-07 was assumed to approximate that with lactase, the same assumptions were used for the second superiority hypothesis (lactase compared with placebo). The same sample size was also considered to be sufficient to reach ≥80% power to demonstrate noninferiority between Bi-07 and lactase, assuming an SD of 0.4 for the within-participant difference between Bi-07 and lactase, a noninferiority margin of 1.25 for the Bi-07:lactase ratio (upper limit of the common bioequivalence range), and a 1-sided α-level of 0.025. The calculations were performed in nQuery Advanced, version 8.2 (Statsols).

##### Efficacy, ancillary, and safety assessments

The per-protocol (PP) population was the primary analysis population, whereas the intention-to-treat (ITT) population was used to support the primary evaluations. To qualify for the stringent PP population, participants must have followed the study protocol without any major violations, as detailed during the BDR (**Supplemental Methods 4** lists the participant classification criteria). The primary efficacy variable in the clinical trials was the iAUC of BHC (ppm · h) during the 6-h acute lactose challenge.

There were 3 primary hypotheses (the 3 pairwise treatment comparisons of the primary endpoint) that were tested statistically: superiority of Bi-07 to placebo, superiority of lactase to placebo, and noninferiority of Bi-07 compared with lactase. The 3 comparisons were evaluated in a 2-step hierarchical order, wherein the superiority of Bi-07 compared with placebo was tested first. In the second step, the superiority of lactase compared with placebo and the noninferiority of Bi-07 compared with lactase were tested simultaneously. Owing to this hierarchical approach, no correction for multiplicity was needed (35, 36).

The primary efficacy variable (iAUC) was analyzed on a ln-transformed scale. iAUC values were calculated using the linear trapezoidal rule. A linear mixed model that was appropriate for the 3-period crossover design was fitted. If an iAUC value was 0 (i.e., all concentrations were at or below the mean baseline), the concentration at 180 min was imputed as the mean baseline value + 0.5, and the iAUC was calculated with this imputed concentration. In the case of individual missing time points for breath hydrogen measurements, it was assumed that the missing values fell linearly between existing data points.

The model included baseline BHC as a covariate; the main effects of sequence, period, and treatment as fixed effects; and participant within sequence as a random effect. The 95% CI for the difference between active treatments and placebo and the corresponding *P* value were obtained from the model and used to evaluate the superiority hypotheses. Using anti-log transformation, the CIs were converted to inferences based on the geometric LSM ratios. A 2-sided 95% CI for the difference (Bi-07 − lactase) was used to evaluate the noninferiority hypothesis. Noninferiority was considered as demonstrated if the CI for the iAUC ratio was completely below the noninferiority margin of 1.25 (the upper limit of the common range for acceptance of bioequivalence). The presence and absence of carryover effects were evaluated separately by adding a first-order carryover effect to the defined statistical model (37). The normality of studentized model residuals was confirmed visually and using normality tests, to detect deviations from model assumptions.

The C_max_ of breath hydrogen was analyzed with similar statistical modeling techniques as in the primary analysis. The cumulative BHC was analyzed with an RM-ANCOVA model, with period-wise mean baseline as a covariate and treatment, period, sequence, time point, and the interaction between treatment and time point as fixed effects. Participant nested within sequence was the random participant effect. The change from period-wise mean baseline concentration was the response in the model.

The period-wise maximum severity of abdominal pain, flatulence, bloating, and nausea was analyzed using mixed-effects cumulative logit models. The occurrence of bowel movements was analyzed by conditional logistic regression analysis, and stool consistency was evaluated descriptively. The occurrence of diarrhea was analyzed using a logistic regression model with random intercepts.

The difference between treatments in mean baseline fasting BHC was analyzed using a similar linear mixed model as in the primary analysis for iAUC, excluding the baseline covariate. Breath carbon dioxide concentration was analyzed using an RM-ANCOVA model that was similar to what was applied for BHC. Breath methane concentration was dichotomized and analyzed using a logistic regression model with random intercepts; this change in the SAP was made before the data were unblinded. Bi-07 concentrations and the SNP variants were reported descriptively without undergoing a statistical analysis. Treatment responders were analyzed based on the iAUC values of the breath hydrogen measurements. A responder was predefined as having experienced at least a 20% decrease in breath hydrogen iAUC compared with the value at screening. Responder data were analyzed by conditional logistic regression. The regression model included treatment and period as fixed terms and participant as the random term.

All AEs were tabulated per MedDRA® [the Medical Dictionary for Regulatory Activities terminology is the international medical terminology developed under the auspices of the International Council for Harmonisation of Technical Requirements for Pharmaceuticals for Human Use (ICH), version 21.0.] by severity and causality. Pretreatment AEs, treatment-emergent AEs (TEAEs), SAEs, and AEs that led to discontinuation were reported separately. The incidence of GI disorders that were at least moderate in severity was analyzed by conditional logistic regression. The regression model included treatment and period as fixed terms and participant as the random term. Safety laboratory tests at the screening were summarized descriptively, and all abnormal findings were reported. All statistical calculations were performed by 4Pharma Ltd. using SAS version 9.4 (SAS Institute Inc.).

## Results

### Lactase activity in an upper GIT simulation model

In the first experiment, which was performed with various bacterial strains and mixtures in batch culture, the mean ± SD lactase activity levels (nmol · g^−1^ · min^−1^) were similar in most cases: Bi-07: 1.146 ± 0.005; NCFM: 1.139 ± 0.007; Lb-87: 1.166 ± 0.012; St-21: 1.087 ± 0.011; Bulk Set Y: 1.115 ± 0.007; YO-MIX 601: 1.099 ± 0.007; and YO-MIX 401: 1.154 ± 0.009. In contrast, HOWARU Dophilus had low activity (0.033 ± 0.000).

In subsequent experiments, the enzymatic activity in the stomach and duodenal stages of the simulated upper GIT was determined in a subset of the strains and mixtures from the first experiment. In these simulations, pure β-galactosidase had similar mean ± SD activity in the stomach and duodenal stages: 1.832 ± 0.006 and 1.678 ± 0.004 nmol · g^−1^ · min^−1^, respectively. HOWARU Dophilus had low activity in both stages (stomach: 0.215 ± 0.003; duodenum: 0.212 ± 0.0.001), whereas YO-MIX 601 showed higher activity in the duodenal stage (stomach: 0.281 ± 0.003; duodenum: 1.765 ± 0.007). Bi-07 had high activity in the stomach (1.465 ± 0.005) and duodenal stages (2.475 ± 0.006), rendering it the most promising of the strains and mixtures; thus, it was selected for further in vitro testing and eventually used in the clinical trials.

### Lactase activity in milk

**FIGURE 2:**
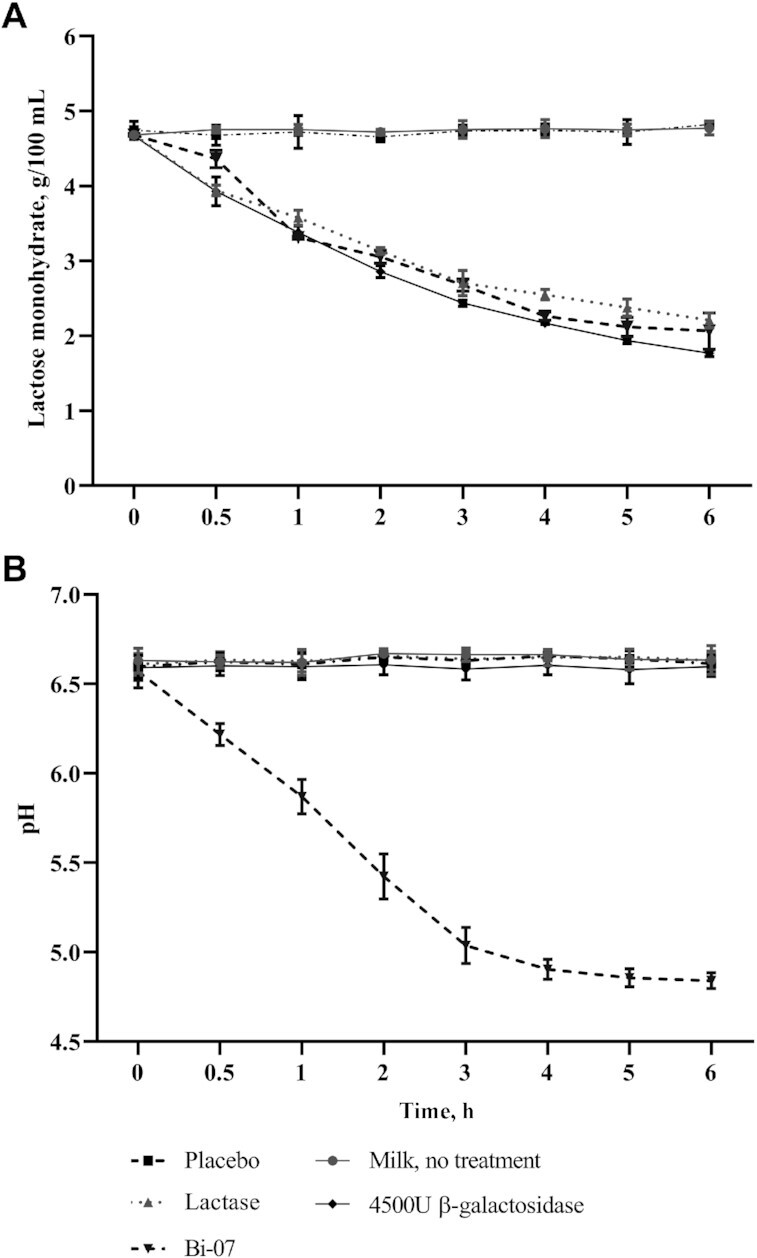
Lactase activity measurement of untreated milk (blank control), placebo (maltodextrin; negative control), lactase (commercial lactase), Bi-07, and 4500 U laboratory-grade β-galactosidase (positive control) in milk. Results are presented as mean ± SD from 3 separate experiments. (A) Amount of lactose monohydrate (g/100 mL) during the 6-h treatment for each of the studied samples. (B) Change in pH during the 6-h treatment in each of the studied samples. Bi-07, *Bifidobacterium animalis* subsp. *lactis* Bi-07.

Bi-07 effected a decrease in the amount of lactose comparable with those of the commercial lactase and laboratory-grade β-galactosidase (**Figure 2**A), whereas with the untreated milk (blank control) and maltodextrin (used as placebo in the clinical trials), there was no change in lactose amounts during the 6 h of measurements.

Only Bi-07 had an impact on pH (Figure 2B), decreasing it from 6.5 to just below 5. There was a significant difference in lactose content over time (*P* < 0.001) in the Bi-07 and lactase treatments compared with placebo but no significant difference (*P* = 0.073) was observed between the Bi-07 and lactase treatments.

### Clinical trials

#### Demographics and disposition of participants

**FIGURE 3:**
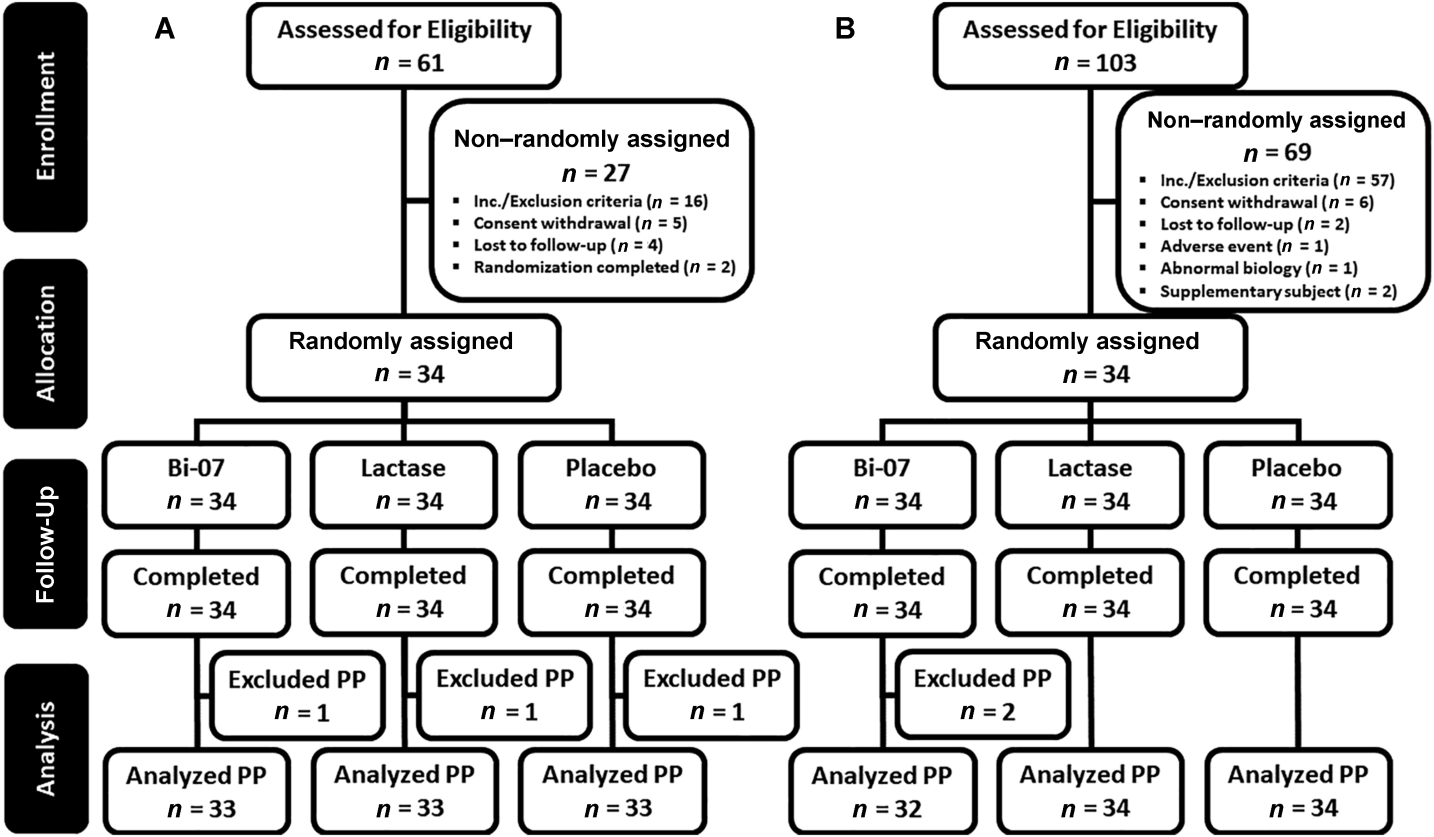
CONSORT flow diagrams of the 2 clinical trials, Booster Alpha (A) and Booster Omega (B). Randomization completed: participant was withdrawn from the study after randomization; abnormal biology: screening assessments showed biologically relevant abnormal blood test values, and the participant was not included in the study; supplementary participant: any potential participant who was prescreened for the study but not randomly assigned, because the required number of randomly assigned participants was obtained before the randomization visit. Bi-07, *Bifidobacterium animalis* subsp. *lactis* Bi-07; PP, per-protocol.

**Table 1** lists the demographics of the clinical trial participants, and **Figure 3** shows their disposition. In Booster Alpha, of the 34 randomly assigned study participants, 1 was excluded from the PP population owing to smoking. In Booster Omega, all 34 randomly assigned participants were included in the PP population, but the results of 2 participants from 1 study visit were excluded owing to the potential unreliability of the outcome assessment as a result of vomiting shortly after consumption of the IP.

**TABLE 1:** Demographics of the intention-to-treat population in Booster Alpha and Omega

		Booster Alpha	Booster Omega
Variable	Statistics	(*n* = 34)	(*n* = 34)
Age, y	*n*	34	34
	Mean ± SD	37.7 ± 11.06	40.2 ± 11.28
	Median	34.5	38.8
	Min, max	25.0, 60.0	25.0, 60.0
Sex			
Male	*n* (%)	15 (44.1)	11 (32.4)
Female	*n* (%)	19 (55.9)	23 (67.6)
Childbearing potential?			
Yes	*n* (%)	16 (84.2)	19 (82.6)
No	*n* (%)	3 (15.8)	4 (17.4)
Height, cm	*n*	34	34
	Mean ± SD	172.0 ± 9.85	166.1 ± 7.45
	Median	172.0	165.0
	Min, max	155.0, 193.0	154.0, 183.0
Weight, kg	*n*	34	34
	Mean ± SD	71.2 ± 13.21	66.1 ± 13.44
	Median	69.3	64.7
	Min, max	47.7, 98.2	47.0, 109.6
BMI, kg/m^2^	*n*	34	34
	Mean ± SD	23.9 ± 3.02	23.8 ± 3.93
	Median	23.9	23.2
	Min, max	18.0, 29.3	18.2, 35.0

#### Primary outcomes

**FIGURE 4:**
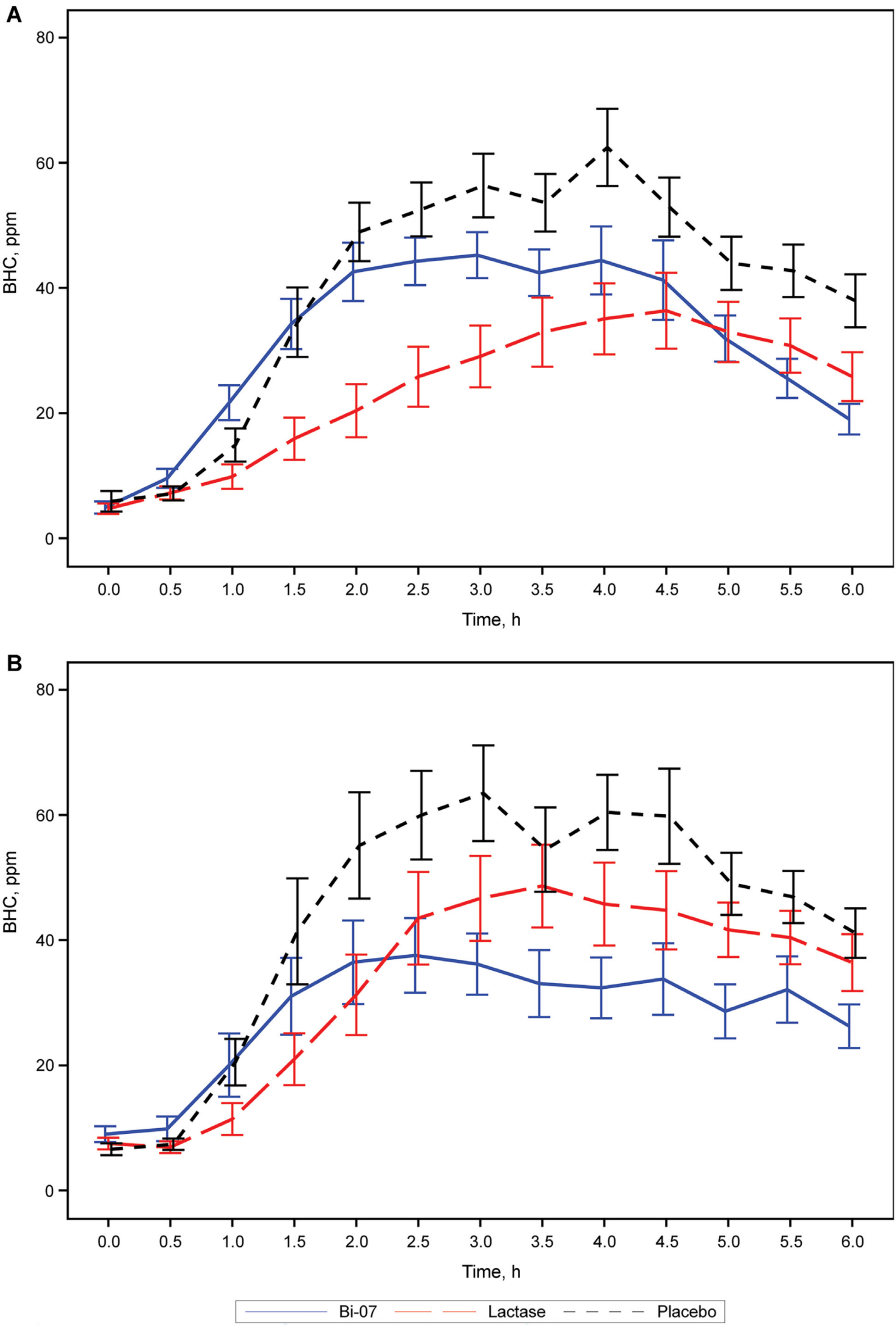
Mean BHC by treatment in per-protocol population during the 6-h lactose challenge in both clinical trials. (A) Booster Alpha (*n* = 33 for each treatment) and (B) Booster Omega (*n* = 32 for Bi-07 and *n* = 34 for lactase and placebo). The SEM at each time point is shown as vertical line segments. BHC, breath hydrogen concentration; Bi-07, *Bifidobacterium animalis* subsp. *lactis* Bi-07.

The primary variable was the iAUC of BHC (ppm · h) during the 6-h acute lactose challenges at V3–V5. **Figure 4** shows the BHC measurements. In Booster Alpha, the superiority of Bi-07 compared with placebo was demonstrated, as evidenced by the geometric LSM ratio of 0.462 (95% CI: 0.249, 0.859), with a *P* value of 0.016. Because the first superiority hypothesis was shown to be true, the 2 other primary hypotheses were then tested simultaneously. The superiority of lactase over placebo was clearly observed, based on the geometric LSM ratio of 0.190 (95% CI: 0.102, 0.365) and *P* value of <0.001. The noninferiority of Bi-07 compared with lactase was not shown, because the upper limit of the 95% CI for the geometric LSM ratio (4.550) was above the noninferiority margin of 1.25 (LSM ratio: 2.431; 95% CI: 1.299, 4.550; *P* = 0.006)—i.e., Bi-07 was superior to placebo but inferior to lactase, based on the iAUC values for BHC. The sensitivity analysis of the ITT population (*n* = 34) supported the primary result, the findings of which were similar to those for the PP population.

The primary model showed a significant sequence effect (*P* = 0.022), which was further examined using descriptive statistics of iAUC values as well as visually (**Supplemental Figure 1**). The sequence effect was explained primarily by 1 of the sequences (placebo–Bi-07–lactase) effecting smaller mean concentrations than the other 5. Further, lactase treatment had the lowest mean iAUC value in all 6 sequences, and Bi-07 treatment elicited numerically lower values than placebo in 5 of the 6 sequences. The anomalous sequence (placebo–Bi-07–lactase) also included 2 outlying participants, for whom BHC concentrations decreased from the mean baseline measurement after lactase treatment. The resulting iAUC was 0, and the imputed iAUC approached 0. Thus, these 2 values deviated from the distribution of iAUC values. A sensitivity analysis, from which these imputed values were omitted, supported the primary analysis but yielded a narrower CI for the geometric LSM ratio between Bi-07 and lactase (LSM ratio: 2.135; 95% CI: 1.321, 3.452; *P* = 0.003).

A significant carryover effect (*P* = 0.004) was observed. In addition to including it in the modeling of the data to enable appropriate interpretation of the data, and performing thorough inspection of the individual BHC data, it was examined further using the Bi-07 quantification data from fecal samples that were collected just before V3 and at the end of each washout period. Six participants had measurable Bi-07 concentrations in their feces in 1 period during the study (more details on the fecal Bi-07 analyses are given in the “Ancillary assessments” section). A similar linear mixed-effects model was fitted to the data as in the primary analysis, except that the amount of Bi-07 was added as a covariate to the model. Because most measurements on the quantity of Bi-07 were 0 (∼95%), quantity did not influence the treatment comparisons (*P* = 0.789). However, considering the individual data of the 6 participants with measurable Bi-07 in their fecal samples, it appears that in sequences in which the placebo was preceded by Bi-07, the concentration curves for the placebo were low, indicating a carryover effect of Bi-07 into the placebo period.

In Booster Omega, the superiority of Bi-07 compared with placebo was demonstrated, as evidenced by the geometric LSM ratio of 0.227 (95% CI: 0.095, 0.543), with a *P* value of 0.001. Based on the first hypothesis being proven, the other 2 primary hypotheses were tested simultaneously. The noninferiority of Bi-07 compared with lactase was observed (LSM ratio: 0.460; 95% CI: 0.193, 1.096; *P* = 0.079), given that the upper limit of the 95% CI of the geometric LSM ratio (1.096) was below the noninferiority margin of 1.25—i.e., Bi-07 was superior to placebo and noninferior to lactase, based on BHC iAUC values. Notably, the superiority of lactase over placebo was not seen, with a geometric LSM ratio of 0.493 (95% CI: 0.210, 1.156) and *P* value of 0.102.

Further, in Booster Omega, the primary model also revealed a significant sequence effect (*P* = 0.008) but no carryover effect. Two of the treatment sequences (Bi-07–lactase–placebo and placebo–lactase–Bi-07) elicited lower mean BHCs than the other 4, explaining the sequence effect (Supplemental Figure 1). Further, Bi-07 treatment effected the lowest mean iAUC value in 3 of 6 sequences (Bi-07–lactase–placebo, placebo–lactase–Bi-07, lactase–placebo–Bi-07) and comparable values with lactase in the other 3 (Bi-07–placebo–lactase, lactase–Bi-07–placebo, and placebo–Bi-07–lactase). Bi-07 also had higher values than placebo in all 6 sequences. **Supplemental Table 1** shows the iAUC descriptive statistics and statistical analysis, and **Supplemental Table 2** describes the BHC results by treatment and time point.

#### Secondary outcomes for BHC

In Booster Alpha, the differences in C_max_ between treatments were similar to those for iAUC. Bi-07 and lactase were superior to placebo, based on their lower C_max_ values. On average, the C_max_ was ∼20 ppm lower with Bi-07 and ∼40 ppm lower with lactase than with placebo. Lactase was also superior to Bi-07 (*P* = 0.006). As in the primary analysis, a significant carryover effect (*P* = 0.050) was observed in the analysis of C_max_. In the analysis of cumulative BHC all 3 treatments differed significantly from each other, wherein the placebo yielded the highest mean concentrations, whereas lactase effected the lowest. The statistics for BHC C_max_ are shown in **Supplemental Table 3**, and those for cumulative BHC are listed in **Supplemental Table 4** for both studies.

In Booster Omega, in the analysis of C_max_, Bi-07 and lactase were superior to placebo. On average, the maximum concentration was ∼40 ppm lower with Bi-07 and ∼25 ppm lower with lactase than with placebo. Bi-07 had a lower C_max_ than lactase, but this finding did not achieve superiority (*P* = 0.080). The analysis of cumulative BHC yielded similar results as for C_max_, but in the former, all 3 treatments differed significantly from each other—the placebo had the highest mean concentrations and Bi-07 had the lowest.

#### Secondary outcome assessments: GI symptoms and bowel function

In Booster Alpha, there were no significant differences in any GI symptom between Bi-07 and placebo. With lactase, participants tended to experience fewer or milder symptoms of abdominal pain and flatulence than with the other treatments. The likelihood of experiencing higher-severity abdominal pain (based on the participant-wise maximum severity that was experienced during each challenge) with lactase treatment was 0.32 times that with Bi-07 (*P* = 0.033) and placebo (*P* = 0.036), and the odds of experiencing higher-severity flatulence with lactase were 0.29 and 0.25 times those with Bi-07 (*P* = 0.015) and placebo (*P* = 0.007), respectively. There were no significant differences in the other symptoms between the 3 groups.

In Booster Omega, except for symptoms of nausea, there were no differences between the 3 treatments. With regard to nausea, participants experienced symptoms with greater frequency and severity during Bi-07 treatment than for placebo and lactase. The odds of experiencing higher-severity nausea with Bi-07 were 4-fold and 7-fold higher than with placebo (*P* = 0.005) and lactase (*P* < 0.001).

**FIGURE 5:**
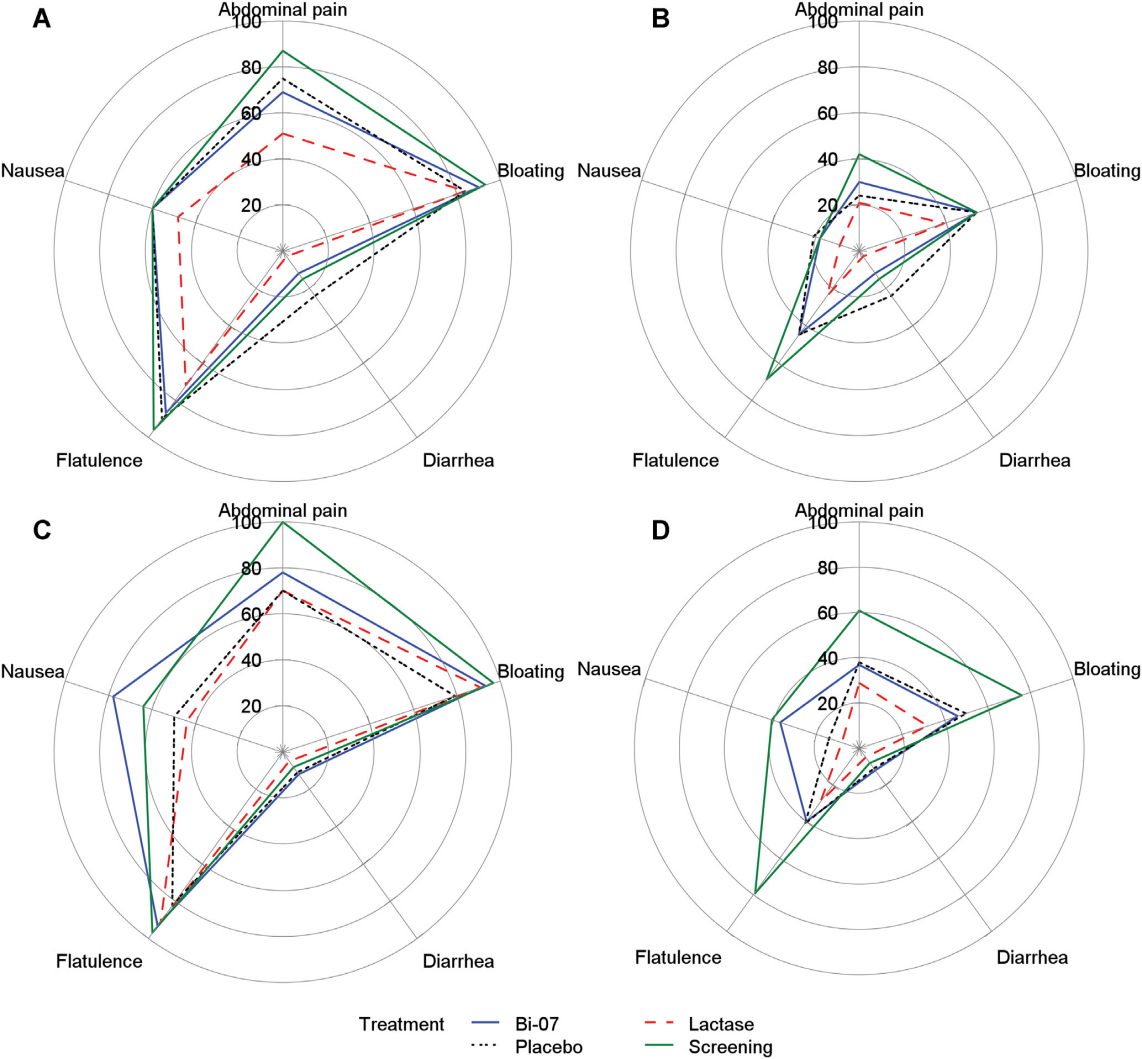
Radar plots of the proportion (mean percentage) of participants in the per-protocol population (Booster Alpha: *n* = 33 for each treatment; Booster Omega: *n* = 32 for Bi-07 and *n* = 34 for lactase and placebo) with gastrointestinal symptoms in both clinical trials. (A) Participants having at least mild symptoms in Booster Alpha, (B) participants having at least moderate symptoms in Booster Alpha, (C) participants having at least mild symptoms in Booster Omega, and (D) participants having at least moderate symptoms in Booster Omega. Bi-07, *Bifidobacterium animalis* subsp. *lactis* Bi-07.

In Booster Alpha, no participants experienced vomiting during the site visits, compared with 2 in Booster Omega. During the BDR, these events were considered major protocol violations owing to the potential for improper ingestion of the IP—1 of these participants vomited immediately after and the other 3 h after consumption of the IP preparation, prompting the measurements from those visits to be excluded from the PP population. **Figure 5** summarizes the proportions of participants who experienced at least mild (Figure 5A, C) or moderate (Figure 5B, D) GI symptoms during the screening period and in each treatment period in both studies. **Supplemental Table 5** lists descriptive statistics of the GI symptoms.

In Booster Alpha, postdose bowel movements during the lactose challenge were reported for 19 participants who received placebo, 19 who consumed Bi-07, and 11 who were administered lactase. Results were significant for lactase compared with placebo (*P* = 0.020) and Bi-07 (*P* = 0.026). The median number of bowel movements per lactose challenge was 1 with Bi-07 and lactase and 2 with placebo. The maximum reported number of bowel movements was 6, for a participant during placebo treatment.

With regard to median stool consistency among participants with bowel movements during the site visits, as assessed per the Bristol Stool Scale, the most frequent categories were Types 3 and 4 for Bi-07 and Type 4 for lactase, indicating normal stool consistency for both treatments. However, Type 7, indicating severe diarrhea, was most commonly observed with placebo. Normal (Type 3 or 4) median stool consistency was observed in 42.1% of participants who had bowel movements during Bi-07 treatment, compared with 36.4% during lactase treatment and 21.1% with placebo. Softer stools were observed with Bi-07 than with lactase. The differences in stool consistency (with softer stools indicating greater lactose malabsorption) are consistent with the disparities in the effects on BHC between the 2 treatments, wherein lactase had a larger effect on lactose maldigestion.

In Booster Omega, postdose bowel movements during the lactose challenge were reported for 14 participants who were given placebo, compared with 8 each who received Bi-07 and lactase. The differences between treatments were not significant. The median number of bowel movements was 1 for lactase and placebo and 1.5 for Bi-07. The maximum reported number of bowel movements was 5, for 3 participants during the placebo treatment. Stool consistency varied widely between treatments, ranging from Type 1 to Type 7. There was no association between treatment and stool consistency in the participants with bowel movements during their visits.

#### Ancillary assessments

There were no differences in baseline BHC or a methane concentration of ≥10 ppm between treatments in either trial. In Booster Omega, the concentration of carbon dioxide with Bi-07 was slightly higher than with lactase (LSM: 0.012; 95% CI: 0.003, 0.022; *P* = 0.009). For most participants, methane concentrations were near 0 throughout the observation period with all treatments, but 10%–15% of participants in Booster Alpha and ∼30% in Booster Omega had increased concentrations. High quantities of methane are associated with slow GI transit times (38).

Bi-07 was quantified from the participants’ fecal samples, collected within 48 h before V3, V4, and V5. No Bi-07 was detected in most samples in either study. In Booster Alpha, 6 samples had a weak signal, 3 of which were within the quantifiable range of the assay (>5.79 × 10^7^ genomes/g feces; the highest observed quantity was 1.52 × 10^9^ genomes/g feces). Each of the 6 samples had been preceded by consumption of Bi-07. In Booster Omega, 13 samples gave a signal, 10 of which were quantifiable (>1.29 × 10^8^ genomes/g feces; the peak value was 6.40 × 10^9^ genomes/g feces). Notably, in 4 of the samples from 3 participants, the positive sample was not preceded by treatment with Bi-07; 1 such participant had a low Bi-07 signal in all samples, despite consuming Bi-07 solely as the final treatment in the sequence. This result suggests that this participant consumed restricted foods or dietary supplements during the study or harbored closely related bifidobacteria (e.g., *B. animalis* subsp. *lactis*) in the gut microbiota. However, because the BHC assessment during the screening confirmed the participant as having lactose maldigestion and because the concentration of *B. lactis* was low, the participant was not excluded from the PP population.

In both studies, the SNP profiles in the lactase gene were homogeneous between participants. In Booster Alpha, 33 participants in the ITT population had the following SNP profile: -13907 C/C, -13910 C/C, -13915 T/T, and -14010 G/G. One participant had a variable genotype, comprising -13915 G/G and -14010 T/T, the former of which has been linked to lactase persistence (i.e., functioning lactase) (30); the latter has not been described, but it is possible that this variant also affects the function of this enzyme. However, because the participant had 2 SNP variants that have been linked to lactase nonpersistence in clinical and in vitro studies (30), they are likely to have been sufficient in inhibiting transcription of the lactase gene and thus causing lactose maldigestion.

In Booster Omega, 33 participants had the following SNP profile: -13907 C/C, -13910 C/C, -13915 T/T, -14010 G/G, and -22018 G/G. One participant had a slightly different genotype, harboring SNPs -13910 C/T and -22018 G/A, indicating heterozygosity for these lactase nonpersistence–linked SNPs. However, owing to the homozygosity of the other lactase deficiency–linked SNPs, it is likely that the participant has lactase deficiency. Notably, 2 SNPs, -13910 and -22018, are in linkage disequilibrium—i.e., they vary simultaneously (30). Based on these results, we concluded that all participants in both studies were lactase deficient.

With regard to treatment response, in Booster Alpha, 72.7%, 48.5%, and 21.2% of participants were responders to lactase, Bi-07, and placebo, respectively. The odds of being a responder to lactase and Bi-07 were 10.7-fold (*P* < 0.001) and 3.4-fold higher (*P* = 0.049) than for placebo, respectively. Lactase did not achieve statistical significance against Bi-07 (OR: 0.3, *P* = 0.068). In Booster Omega, 78.1%, 67.6%, and 41.2% responded to Bi-07, lactase, and placebo, respectively. The odds of being a responder to Bi-07 were 7.7-fold higher (*P* = 0.002) than for placebo. Bi-07 was associated with the highest response rates but did not significantly differ from lactase in this regard (OR: 2.0, *P* = 0.260). Lactase had significantly higher response rates than placebo (OR: 3.8, *P* = 0.021).

#### Safety assessments

In Booster Alpha, there were 121 pretreatment AEs during the screening visit (with a lactose challenge) and 317 TEAEs throughout the study. Of the TEAEs, 111 were reported during the Bi-07 treatment (97.1% of participants, i.e., 33 participants of the total population), compared with 115 with placebo (97.1% of participants, *n* = 33) and 91 with lactase (91.2%, *n* = 31). A total of 315 TEAEs were considered to have been related to the treatment, likely due to the study design—i.e., consumption of the IP with the source of lactose by lactase-deficient participants. Thirty-one of the TEAEs were severe, 12 of which occurred with Bi-07, compared with 14 with placebo and 5 with lactase. A higher proportion of participants experienced AEs that were at least moderate in severity with Bi-07 than with the other treatments (73.5% with Bi-07, 50.0% with lactase, and 67.6% with placebo) and the difference was statistically significant (*P* = 0.031) between Bi-07 and lactase. None of the AEs led to discontinuation of the study product, and no SAEs were reported. All but 1 (dizziness) of the pretreatment AEs and TEAEs were confined to the GIT. The most common AEs, both pretreatment and TEAEs, were abdominal distension, flatulence, and abdominal pain.

In Booster Omega, 149 pretreatment AEs and 303 TEAEs were reported. Of the TEAEs, 114 arose during the Bi-07 treatment (100% of participants, *n* = 34), compared with 93 during placebo (94.1% of participants, *n* = 32) and 96 during lactase (94.1%, *n* = 32). A total of 268 TEAEs were classified as possibly or probably related to the treatment, again most likely due to consumption of the product coinciding with the lactose challenge. Of the TEAEs, 31 were severe, 14 of which occurred with Bi-07, compared with 11 with placebo and 6 with lactase. Further, 61.8%, 44.1%, and 58.8% of participants who consumed Bi-07, lactase, and placebo, respectively, experienced GI disorders that were at least moderate in severity; no significant differences were observed between the treatments. The most common TEAEs were flatulence, abdominal distension, abdominal pain, and nausea. All but 5 (1 case of dizziness and 4 cases of headache) pretreatment AEs and TEAEs were confined to the GIT. One episode of gastroenteritis occurred between V3 and V4, which was a protocol violation, but because it was short in duration (1 d) and because the washout period could be extended to 22 d to avoid possible effects on the results for V4, the participant was not excluded from the study. None of the AEs led to discontinuation, and no treatment-emergent SAEs were reported. One prescreened participant experienced an SAE (fracture of the right collarbone) before beginning the treatment and thus withdrew from the study.

All participants in both studies had normal results on the physical examination. Safety laboratory measurements indicated abnormal values for certain participants, but none was defined as an AE or required withdrawal from the study.

## Discussion

In this study, we examined whether consumption of Bi-07 aids lactose maldigestion. The lactase activity of Bi-07 was compared with *L. acidophilus* NCFM (grown in the presence or absence of dairy components), yogurt starter cultures, as well as *L. bulgaricus* and *S. thermophilus. L. acidophilus* NCFM was selected based on data indicating that it relieves symptoms of lactose intolerance (39–41). Yogurt starter cultures, *L. bulgaricus*, and *S. thermophilus* were included in the study because the consumption of yogurt with live cultures improves the digestion of lactose in people with lactose maldigestion (42).

As expected, only the strains and mixtures that were cultured in the presence of dairy components had high lactase activity. Similar results were obtained in the upper GIT simulation. Bi-07 had higher lactase activity than the other strains. The amount of Bi-07 was adjusted to correspond to 4500 FCC units of lactase activity, a quantity of lactase that is generally used in supplements and is the minimum amount of lactase with a health claim in the European Union (18). Thus, 2.40 × 10^12^ CFU Bi-07 and 2.34 × 10^12^ CFU Bi-07 were the amounts that were examined in Booster Alpha and Booster Omega, respectively.

Clinical trials on the effects of probiotics on lactose maldigestion have often been chronic studies with low daily probiotic doses (16, 43, 44). These studies have shown effects on GI symptoms (e.g., lactose intolerance) but to a limited extent on lactose maldigestion, based on BHCs (16, 44). The trial setting of our study differs because its aim was to study the effects of Bi-07 after acute lactose challenge, compared with lactase. Because of the acute nature of the challenge, a crossover design was judged to be most appropriate, because it allowed us to study the effect of several products in the same participants. This property reduces interindividual variability and the total number of participants needed for sufficient statistical power (45). Earlier studies in this area have used a similar trial design (32–34).

The results on the primary outcome differed between the 2 clinical trials, showing greater efficacy of lactase in Booster Alpha, whereas Bi-07 was more efficacious in Booster Omega. This disparity is likely due to the differing sources of lactose between the studies, given that they were otherwise identical, with no apparent contrasts in demographics or lactase nonpersistence–related genetic markers between study populations. In Booster Alpha, in which milk was used as the vehicle, GI transit could have been slowed owing to milk protein coagulation (46), improving the function of lactase in the stomach. In Booster Omega, the water-based lactose solution that was used likely accelerated GI transit, limiting the duration of lactase activity in the stomach. Because the lactase in Bi-07 is intracellular, its activity is likely to be slower than that of freely available lactase. Comparing the BHC measurements with older studies with a similar study design (32–34), the interindividual variation is large across all studies. Further, the BHCs and SDs were similar in our clinical trials compared with the reference studies, and the estimated 35% effect size was consistent with the range in the Booster studies (21.4%–44%).

Lactase relieved lactose intolerance–related symptoms better than Bi-07 in Booster Alpha but not Booster Omega. Bi-07 relieved symptoms similarly to placebo, except for nausea, which increased with Bi-07 in Booster Omega. In the ITT population in Booster Omega, there were 2 cases of vomiting with Bi-07. Across all time points, most symptoms of nausea (40.6% mild, 21.9% moderate, and 3.1% severe; 34.4% had no symptoms) were experienced 1 h after ingestion of the mixture with Bi-07 in the PP population during the lactose challenge (results not shown). Six hours after consumption of the mixture, 9 and 2 participants had mild and moderate symptoms of nausea, respectively, whereas 21 (65.6%) did not experience any symptoms of nausea. Because Bi-07 was used as a pure culture—i.e., without additives—it might have had an unpleasant taste, contributing to the nausea and vomiting. The observation that there was no increase in nausea with Bi-07 in Booster Alpha suggests that its potentially disagreeable flavor was better masked by the milk. This finding indicates that the treatments might not have been concealed as well in Booster Omega as in Booster Alpha. The primary outcome assessments were not affected, because they were based on the nonsubjective measure of BHC.

One of the limitations of the study was the use of an unvalidated GI symptom questionnaire, which was administered owing to a lack of validated questionnaires for this study design at its outset (6, 7). Based on the symptoms that were recorded at the screening challenge, the overall level of symptoms of lactose intolerance was moderate, because the participants were not selected according to a certain threshold of symptom severity. Accordingly, any reduction in symptoms would perhaps be less pronounced than in a population of participants who have been affected to a greater degree. Moreover, a clear placebo effect was observed in both studies.

Notably, there was a significant carryover effect in Booster Alpha, and significant sequence effects were observed in both studies, for both of which there is no clear explanation. However, the carryover effect might have obscured some of the potential of Bi-07 in the Booster Alpha study. In Booster Alpha, in participants in whom Bi-07 was observed in fecal samples 1 wk after its consumption and who consumed placebo after Bi-07, placebo BHC values were lower than expected. This partial digestion of lactose was perhaps attributable to the transient presence of Bi-07. Previous studies have shown that Bi-07 should be cleared from the GIT within 1 wk (47, 48), as is common for many probiotics (49). Based on the present results, longer washout periods should be considered in future studies.

Several studies that have correlated the human genome and gut microbiota composition have linked the abundance of bifidobacteria to lactase deficiency in people who consume milk products, suggesting that bifidobacteria could have a role in the development of lactose tolerance (50). This model thus highlights the potential of bifidobacterial supplements in improving lactose intolerance. Previous long-term intervention and acute lactose challenge studies with bifidobacteria have observed improvements in maldigestion, based on breath hydrogen test and GI symptoms, but higher doses of bacteria, as used in this study, merit examination (16).

Our results have been submitted for a health claim evaluation by the EFSA (51). The EFSA has confirmed that Bi‐07 has been sufficiently characterized, that the claimed effect of an “improvement in lactose digestion” is a beneficial physiologic effect for individuals with lactose maldigestion, that consumption of Bi‐07 (10^12^ CFU) increases lactose digestion in individuals with lactose maldigestion, and that Bi‐07 exhibits lactase activity in vitro. However, the EFSA required a demonstration of an improvement in symptoms of lactose maldigestion, whereas the study was designed to assess the effects on lactose digestion (by breath test) (51). Nevertheless, the Booster Omega study has provided evidence that Bi-07 is noninferior to lactase in improving lactose digestion, the latter of which has been previously approved by the EFSA (18).

We have compared a probiotic with high lactase activity, Bi-07, with a commercial lactase. In assessing its efficacy in alleviating lactose maldigestion, 2 of our clinical trials have shown the superiority of Bi-07 to placebo, and 1 has demonstrated noninferiority to lactase.

## Supplementary Material

nqac264_Supplemental_File

## Data Availability

The data in the article for the clinical trials will not be made available, because the study participants have not been asked for approval to share the data publicly. Other data, the code book, and analytic code will be made available on request.

## Notes

Supported by IFF (DuPont Nutrition and Biosciences at the time of the study).

Authors disclosure: PR, SDF, MM, AI, and ACO are employees of IFF (DuPont Nutrition and Biosciences at the time of the study), which sponsored the study and manufactures and sells *Bifidobacterium lactis* Bi-07. All other authors report no conflicts of interest.

Supplemental Methods 1–4, Supplemental Figure 1, and Supplemental Tables 1–5 are available from the “Supplementary data” link in the online posting of the article and from the same link in the online table of contents at https://academic.oup.com/ajcn/.

Abbreviations used: AE, adverse event; BDR, blind data review; BHC, breath hydrogen concentration; Bi-07, *Bifidobacterium animalis* subsp. *lactis* Bi-07; EC, ethical committee; EDC, electronic data capture; EFSA, European Food Safety Authority; FCC, Food Chemicals Codex; GI, gastrointestinal; GIT, gastrointestinal tract; iAUC, incremental area under the curve; IP, investigational product; ITT, intention-to-treat; Lb-87, *Lactobacillus delbrueckii* subsp. *bulgaricus* Lb-87; LSM, least square mean; NCFM, *Lactobacillus acidophilus* NCFM; PI, principal investigator; PP, per-protocol; ppm, parts per million; RM, repeated-measures; SAE, serious adverse event; SAP, statistical analysis plan; SNP, single-nucleotide polymorphism; St-21, *Streptococcus thermophilus* St-21; TEAE, treatment-emergent adverse event.
