# Supplementary material for: Bifidobacterium animalis subsp. lactis Bi-07 supports lactose digestion in vitro and in randomized, placebo- and lactase-controlled clinical trials
Source: Am J Clin Nutr. 2022 Sep 23;116(6):1580–94. doi: 10.1093/ajcn/nqac264 (PMC9761758; doi:10.1093/ajcn/nqac264)
Supplement: nqac264_Supplemental_File [file nqac264_supplemental_file.docx]

**Supplementary methods, tables and figures**

***Bifidobacterium animalis* subsp. *lactis* Bi-07 supports lactose digestion *in vitro* and in randomized, placebo and lactase -controlled clinical trials**

**by Rasinkangas *et al.***

***Contents***

**Supplementary methods 1: Inclusion and exclusion criteria used in the clinical studies *p. 2***

**Supplementary methods 2: Gastrointestinal symptom questionnaire *p. 6***

**Supplementary methods 3: Materials and methods for fecal sampling and quantitative PCR of *B. lactis* Bi-07 *p. 8***

**Supplementary methods 4: Criteria of Subject Classification *p. 9***

**Supplementary tables 1: Analysis of iAUC_0-360min_ of BHC (ppm.h) *p. 10***

**Supplementary tables 2: Breath hydrogen concentrations (BHC, ppm) by treatment and time point (PP) *p. 12***

**Supplementary tables 3: Analysis of C_max_ of breath hydrogen concentration (BHC, ppm) *p.16***

**Supplementary tables 4: Cumulative BHC analysis *p. 18***

**Supplementary tables 5: Descriptive statistics of gastrointestinal symptoms *p. 20***

**Supplementary figures 1: Treatment sequence effect assessment *p. 24***

**Supplementary methods 1: Inclusion and exclusion criteria used in the clinical studies**

***Booster Alpha:***

**Inclusion criteria:**

1. Voluntary, written, informed consent to participate in the study

2. Agreement to comply with the protocol and study restrictions

3. Healthy females and males of age 25 to 60 years (inclusive)

4. Self-declared or medically diagnosed lactose intolerance

5. Increase of more than 20 ppm in breath hydrogen within 3 h from the baseline fasting breath hydrogen value at V2

6. Participants who agree to maintain their usual dietary habits throughout the trial period

7. Participants who agree not to consume probiotics, prebiotics, fermented milk, and/or yogurt containing probiotics during and two weeks before Visit 3 (2 weeks after pre-screening visit)

8. Females of child-bearing potential who agree to use a medically approved methods of birth control

9. Ability of the participant (in the investigator’s opinion) to comprehend the full nature and purpose of the study including possible risks and side effects

10. Covered by Health Insurance System and / or in compliance with the recommendations of National Law in force relating to biomedical research

**Exclusion criteria:**

1. Gastrointestinal disorder or disease (e.g. Crohn’s disease, ulcer, irritable bowel syndrome (IBS), celiac disease, small intestinal bacterial overgrowth (SIBO), pancreatitis and disorders affecting gastrointestinal motility)

2. Diagnosed type 1 or type 2 diabetes

3. Prior abdominal surgery that, in the opinion of the investigator, may present a risk for the participant or affect study results

4. Ongoing or recent (last 1 months) antibiotic treatment

5. Use of laxatives or drugs known to affect gut motility 1 month preceding the screening visit and during the study

6. Ongoing or recurrent use of proton pump inhibitors

7. Colonoscopy within 3 months before screening

8. History of recurrent colon cleansing and/or a colon cleansing within 3 months before screening

9. Gastrointestinal infection within 1 month before screening or during the trial

10. Clinically significant underlying systemic illness that may preclude the participant’s ability to complete the trial or that may affect the study outcomes (e.g. bowel cancer, prostate cancer, terminal illness)

11. History of diagnosed coronary heart disease/cardiovascular disease or artificial heart valve

12. Any obstructive or restrictive respiratory syndrome/disease that may impact breath test (e.g. asthma or chronic obstructive pulmonary disease (COPD))

13. Use of tobacco, snuff, nicotine and e-cigarette

14. History of or current abuse of drugs, alcohol or medication (self-reported)

15. Self-declared use of illicit drugs

16. Pregnant or lactating female, or pregnancy planned during study period

17. Participants under administrative or legal supervision

18. Participation in another study with any investigational product within 60 days of screening

19. Abnormal values in safety blood tests at V1 i.e. clinically significant or >2x upper limit of normal, unless the deviation is justified by a previously known not clinically relevant condition (e.g. Gilbert's syndrome)

20. Other reasons that, in the opinion of the investigator, make the participant unsuitable for enrolment

***Booster Omega:***

**Inclusion criteria:**

1. Voluntary, written, informed consent to participate in the study

2. Agreement to comply with the protocol and study restrictions

3. Healthy females and males of age 25 to 60 years (inclusive)

4. Self-declared, suspected or medically diagnosed lactose intolerance

5. Increase of more than 20 ppm in breath hydrogen within 3 h from the baseline fasting breath hydrogen value

6. Participants who agree to maintain their usual dietary habits throughout the trial period

7. Participants who agree not to consume probiotics, prebiotics, fermented milk, and/or yogurt containing probiotics during the intervention period (Visits 3-5) and two weeks before Visit 3 (2 weeks after screening visit)

8. Females of child-bearing potential who agree to use a medically approved method of birth control

9. Ability of the participant (in the investigator’s opinion) to comprehend the full nature and purpose of the study including possible risks and side effects

10. Covered by Health Insurance System and / or in compliance with the recommendations of National Law in force relating to biomedical research

**Exclusion criteria:**

1. Gastrointestinal disorder or disease (e.g. Crohn’s disease, ulcer, irritable bowel syndrome (IBS), Celiac disease, small intestinal bacterial overgrowth (SIBO), pancreatitis and disorders affecting gastrointestinal motility)

2. Diagnosed type 1 or type 2 diabetes

3. Prior abdominal surgery that, in the opinion of the investigator, may present a risk for the participant or affect study results

4. Ongoing or recent (last 1 month) antibiotic treatment.

5. Use of laxatives or drugs known to affect gut motility 1 month preceding the screening visit and during the study

6. Ongoing or recurrent use of proton pump inhibitors

7. Colonoscopy within 3 months before screening

8. History of recurrent colon cleansing and/or a colon cleansing within 3 months before screening

9. Gastrointestinal infection within 1 month before screening or during the trial

10. Clinically significant underlying systemic illness that may preclude the participant’s ability to complete the trial or that may affect the study outcomes (e.g. bowel cancer, prostate cancer, terminal illness, immunosuppressive disease (e.g. HIV/AIDS)).

11. History of diagnosed coronary heart disease/cardiovascular disease or artificial heart valve

12. Any obstructive or restrictive respiratory syndrome/disease that may impact breath test (e.g. asthma or chronic obstructive pulmonary disease (COPD))

13. Regular use of tobacco, snuff, nicotine and e-cigarette, and possible other inhaled products. Light smokers (less than 5 cigarettes per day) who agree not to smoke during the whole duration of the study could be included.

14. Self-declared history of alcohol abuse (for females: >3 drinks on any single day and >7 drinks per week; for males: >4 drinks on any single day and >14 drinks per week)

15. Self-declared use of illicit drugs within 4 weeks preceding the screening visit

16. Pregnant or lactating female, or pregnancy planned during study period

17. Participants under administrative or legal supervision.

18. Participation in another study with any investigational product within 60 days of screening

19. Clinically significant abnormal values in safety blood tests at screening

20. Other reasons that, in the opinion of the Investigator, make the participant unsuitable for enrolment

21. Who would receive more than 4500€ as indemnities for his/her participation in biomedical research

within the last 12 months, including the indemnities for the present study.

**Supplementary methods 2: Gastrointestinal symptom questionnaire**

**Page 1/2**

**1. Please, rate the severity of abdominal pain you are currently experiencing:**

**None [ ] Mild [ ] Moderate [ ] Severe [ ]**

**2. Please, rate the severity of flatulence you are currently experiencing:**

**None [ ] Mild [ ] Moderate [ ] Severe [ ]**

**3. Please, rate the severity of bloating you are currently experiencing:**

**None [ ] Mild [ ] Moderate [ ] Severe [ ]**

**4a. Please, rate the severity of nausea you are currently experiencing:**

**None [ ] Mild [ ] Moderate [ ] Severe [ ]**

**4b. Have you vomited?**

**Yes [ ] No [ ]**

**5a. Have you experienced bowel movement(s) today?**

**Yes [ ] No [ ]**

**5b. Have you experienced diarrhea today?**

**Yes [ ] No [ ]**

**5c. If yes to 5a and/or 5b, grade the stool consistency and the number of bowel movements in the Bristol Stool Scale table below.**

**Please, use the following codes : 1 = None**

**2 = Mild**

**3 = Moderate**

**4 = Severe**

**5 = Very severe**

| **Stool consistency and number of bowel movements, Question 5:**  **Use the pictures and descriptions below to grade your stools (type 1 to 7) at the visits. Mark also the number of bowel movements (1-7) for each stool type.** | | | | | | | | | |
| --- | --- | --- | --- | --- | --- | --- | --- | --- | --- |
| **Type** | **Image** | **Description** | **Number of bowel movement** | | | | | | |
|  |  |  | **1** | **2** | **3** | **4** | **5** | **6** | **7** |
| Type1 | 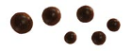 | Separate hard lumps, like nuts (hard to pass) |  |  |  |  |  |  |  |
| Type2 | 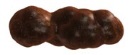 | Sausage-shaped but lumpy |  |  |  |  |  |  |  |
| Type3 | 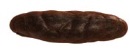 | Like a sausage but with cracks on its surface |  |  |  |  |  |  |  |
| Type4 | 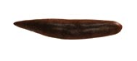 | Like a sausage or snake, smooth and soft |  |  |  |  |  |  |  |
| Type5 | 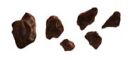 | Soft blobs with clear-cut edges (passed easily) |  |  |  |  |  |  |  |
| Type6 | 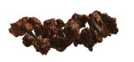k, | Fluffy pieces with ragged edges, a mushy stool |  |  |  |  |  |  |  |
| Type7 | 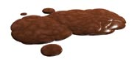 d | Watery, no solid pieces. **Entirely Liquid** |  |  |  |  |  |  |  |

**Page 2/2**

**Supplementary methods 3: Materials and methods for fecal sampling and quantitative PCR of *B. lactis* Bi-07**

Fecal samples were collected from the participants before visits 3, 4 and 5, to be able to detect possible carryover from Bi-07 consumption. The participants collected the samples within 48 h before each study visit at home with sampling and cold delivery kits provided at the clinical site. The samples were stored in home freezers (-18℃) until they were returned frozen to the site at the following study visit, after which they were stored at ≤ -70°C. Quantification of Bi-07 from fecal samples (expressed in Log_genomes_/g feces) was performed in a blinded manner by the Sponsor using absolute quantification qPCR with Taqman Fast Advanced Master Mix (ThermoFisher, Vantaa, FI) as described in Forssten et al.

**Reference**

Forssten SD, Yeung N, Ouwehand AC. Fecal Recovery of Probiotics Administered as a Multi-Strain Formulation during Antibiotic Treatment. Biomedicines. 2020;8(4):83. Published 2020 Apr 9. doi:10.3390/biomedicines8040083

**Supplementary methods 4: Criteria of Subject Classification**

**Definitions of the study populations:**

***Safety population***

Safety population consists of all participants who were randomized and took at least one dose of the study product.

***Intent-to-treat population (ITT)***

Participants are included in the ITT population if they are randomized and receive treatment after randomization.

***Per protocol population (PP)***

PP population is a subpopulation of the ITT population. In order to qualify for the stringent PP population, the participants must have followed the study protocol without any major violations which will be defined in detail during the Blind Data Review process.

Before opening of the randomization code, information on protocol violations was gathered from the locked study databases. ***Protocol violations*** were classified into three categories:

***Category 1) Major that alters the treatment outcome***

***Category 2) Major that do not alter the treatment outcome***

***Category 3) Minor***

Violation graded as major that alters the treatment outcome (Category 1) resulted in excluding all or part of the subjects’ efficacy data from the Per protocol (PP) population.

**Supplementary tables 1: Analysis of iAUC_0-360min_ of BHC (ppm∙h)**

1. Descriptive statistics of iAUC_0-360min_ in Booster Alpha

|  |  |  | | | | | | |  |  |  |
| --- | --- | --- | --- | --- | --- | --- | --- | --- | --- | --- | --- |
|  |  |  |  |  |  |  |  |  |  |  |  |
| Parameter | | | | Statistics | Bi-07 (N=33) | Lactase (N=33) | Placebo (N=33) |  | |  |  |
| iAUC | | | | n | 33 | 33 | 33 |  | |  |  |
| (ppm.h) | | | | Mean (SD) | 172.5 (86.33) | 123.0 (121.20) | 219.4 (101.21) |  | |  |  |
|  | | | | CV(%) | 50.0 | 98.5 | 46.1 |  | |  |  |
|  | | | | Geometric Mean | 145.9 | 46.8 | 192.2 |  | |  |  |
|  | | | | Median | 173.6 | 89.5 | 221.3 |  | |  |  |
|  | | | | Min, Max | 12, 450 | 0, 474 | 28, 431 |  | |  |  |

1. Statistical analysis of iAUC_0-360min_ in Booster Alpha

|  |  |  |  |  |  |
| --- | --- | --- | --- | --- | --- |
|  | | | | | |
| Effect | | Num DF | Den DF | F-value | p-value |
| Sequence | | 5 | 27 | 3.190 | 0.0217 |
| Period | | 1 | 59 | 3.232 | 0.0773 |
| Treatment | | 2 | 59 | 14.055 | <.0001 |
| Baseline | | 1 | 59 | 1.441 | 0.2349 |
| Carryover | | 2 | 59 | 6.012 | 0.0042 |

| **Pairwise comparisons** | | |  |  |  |
| --- | --- | --- | --- | --- | --- |
|  | Bi-07 (N=33) | Lactase (N=33) | | Placebo (N=33) |  |
| Geometric LSM (95 % CI) | 114.8 (70.3, 187.6) | 47.2 (29.0, 77.0) | | 248.3 (152.3, 404.8) |  |
| Comparisons |  |  | |  |  |
| Ratio of Geometric LSM Vs Placebo, 95 % CI | 0.462 (0.249, 0.859) | 0.190 (0.102, 0.356) | |  |  |
| p-value | 0.0156 | <.0001 | |  |  |
| Ratio of Geometric LSM Vs Lactase, 95 % CI | 2.431 (1.299, 4.550) |  | |  |  |
| p-value | 0.0062 |  | |  |  |
| **________________________________________________________________________________________** | | | | | |

1. Descriptive statistics of iAUC_0-360min_ in Booster Omega

|  |  |  | | | | | | |  |  |  |
| --- | --- | --- | --- | --- | --- | --- | --- | --- | --- | --- | --- |
|  |  |  |  |  |  |  |  |  |  |  |  |
| Parameter | | | | Statistics | Bi-07 (N=32) | Lactase (N=34) | Placebo (N=34) |  | |  |  |
| iAUC | | | | n | 32 | 34 | 34 |  | |  |  |
| (ppm.h) | | | | Mean (SD) | 134.7 (120.76) | 162.6 (111.92) | 235.7 (142.80) |  | |  |  |
|  | | | | CV (%) | 89.7 | 68.8 | 60.6 |  | |  |  |
|  | | | | Geometric Mean | 38.8 | 88.4 | 183.2 |  | |  |  |
|  | | | | Median | 127.7 | 160.0 | 226.0 |  | |  |  |
|  | | | | Min, Max | 0, 441 | 0, 368 | 15, 570 |  | |  |  |

1. Statistical analysis of iAUC_0-360min_ in Booster Omega

|  |  |  |  |  |  |  |  |  |  |  |  |  |
| --- | --- | --- | --- | --- | --- | --- | --- | --- | --- | --- | --- | --- |
|  | | | | | | | | |  |  |  |  |
| Effect | | Num DF | Den DF | | F-value | p-value | | |  |  |  |  |
| Sequence | | 5 | 28 | | 3.982 | 0.0075 | | |  |  |  |  |
| Period | | 1 | 61 | | 0.397 | 0.6740 | | |  |  |  |  |
| Treatment | | 2 | 61 | | 5.777 | 0.0050 | | |  |  |  |  |
| Baseline | | 1 | 61 | | 0.356 | 0.5529 | | |  |  |  |  |
| **Pairwise comparisons** | | | | | | | | |  |  |  |  |
|  | | | | | Bi-07 (N=32) | | | Lactase (N=34) | | | Placebo (N=34) |  |
| Geometric LSM (95% CI) | | | | | 40.1 (20.9, 76.8) | | | 87.1 (46.5, 163.4) | | | 176.8 (94.0, 332.6) |  |
| Comparisons | | | | |  | | |  | | |  |  |
| Ratio of Geometric LSM Vs Placebo, 95% CI | | | | | 0.227 (0.095, 0.543) | | | 0.493 (0.210, 1.156) | | |  |  |
| p-value | | | | | 0.0012 | | | 0.1022 | | |  |  |
| Ratio of Geometric LSM Vs Lactase, 95% CI | | | | | 0.460 (0.193, 1.096) | | |  | | |  |  |
| p-value | | | | | 0.0785 | | |  | | |  |  |
| **________________________________________________________________________________________** | | | | | | | | | | | | |
|  | | | | | | | | | | | | |

**Supplementary tables 2: Breath hydrogen concentrations (BHC, ppm) by treatment and time point (PP)**

***a.) Breath hydrogen concentrations by treatment and timepoint in Booster Alpha***

|  |  |  |  |  |  |
| --- | --- | --- | --- | --- | --- |
| Timepoint | | Statistics | Bi-07 (N=33) | Lactase (N=33) | Placebo (N=33) |
| BASELINE (Mean) | | n | 33 | 33 | 33 |
|  | | Mean (SD) | 4.3 (4.42) | 4.6 (4.78) | 4.6 (5.84) |
|  | | Median | 2.0 | 2.0 | 1.5 |
|  | | Min, Max | 0, 16 | 0, 16 | 0, 19 |
|  | |  |  |  |  |
| LACTOSE CHALLENGE | | n | 33 | 33 | 33 |
|  | | Mean (SD) | 4.9 (5.61) | 4.7 (4.80) | 5.9 (9.43) |
|  | | Median | 3.0 | 3.0 | 2.0 |
|  | | Min, Max | 0, 22 | 0, 18 | 0, 40 |
|  | |  |  |  |  |
| 0.5 H POSTDOSE | | n | 33 | 33 | 33 |
|  | | Mean (SD) | 9.6 (8.77) | 7.2 (6.06) | 7.2 (6.39) |
|  | | Median | 6.0 | 5.0 | 6.0 |
|  | | Min, Max | 0, 31 | 1, 20 | 0, 26 |
|  | |  |  |  |  |
| 1 H POSTDOSE | | n | 33 | 33 | 33 |
|  | | Mean (SD) | 21.7 (16.06) | 9.8 (11.25) | 14.9 (15.21) |
|  | | Median | 19.0 | 6.0 | 11.0 |
|  | | Min, Max | 0, 57 | 1, 47 | 1, 76 |
|  | |  |  |  |  |
| 1.5 H POSTDOSE | | n | 33 | 33 | 33 |
|  | | Mean (SD) | 34.2 (23.08) | 15.9 (19.36) | 34.5 (31.95) |
|  | | Median | 34.0 | 8.0 | 28.0 |
|  | | Min, Max | 1, 88 | 0, 76 | 0, 154 |
|  | |  |  |  |  |
| 2 H POSTDOSE | | n | 33 | 33 | 33 |
|  | | Mean (SD) | 42.6 (26.77) | 20.4 (24.40) | 48.9 (26.80) |
|  | | Median | 39.0 | 8.0 | 51.0 |
|  | | Min, Max | 7, 136 | 0, 96 | 0, 108 |
|  | |  |  |  |  |
| 2.5 H POSTDOSE | | n | 33 | 33 | 33 |
|  | | Mean (SD) | 44.2 (21.76) | 25.8 (27.55) | 52.5 (24.72) |
|  | | Median | 44.0 | 15.0 | 52.0 |
|  | | Min, Max | 3, 96 | 0, 107 | 0, 102 |
|  |  |  |  |  |  |
|  |  |  |  |  |  |
|  |  |  |  |  |  |
|  |  |  |  |  |  |
|  |  |  |  |  |  |
|  |  |  |  |  |  |

|  |  |  |  |  |  |
| --- | --- | --- | --- | --- | --- |
| Time point | | Statistics | Bi-07 (N=33) | Lactase (N=33) | Placebo (N=33) |
| 3 H POSTDOSE | | n | 33 | 33 | 33 |
|  | | Mean (SD) | 45.2 (21.08) | 28.5 (28.78) | 56.4 (29.21) |
|  | | Median | 44.0 | 21.0 | 51.0 |
|  | | Min, Max | 1, 91 | 0, 116 | 4, 138 |
|  | |  |  |  |  |
| 3.5 H POSTDOSE | | n | 33 | 33 | 33 |
|  | | Mean (SD) | 42.4 (21.41) | 32.9 (31.73) | 53.6 (26.46) |
|  | | Median | 42.0 | 22.0 | 55.0 |
|  | | Min, Max | 4, 88 | 0, 126 | 6, 106 |
|  | |  |  |  |  |
| 4 H POSTDOSE | | n | 33 | 33 | 33 |
|  | | Mean (SD) | 44.4 (31.20) | 35.1 (32.58) | 62.5 (35.41) |
|  | | Median | 38.0 | 25.0 | 61.0 |
|  | | Min, Max | 3, 163 | 0, 145 | 10, 159 |
|  | |  |  |  |  |
| 4.5 H POSTDOSE | | n | 33 | 33 | 33 |
|  | | Mean (SD) | 41.2 (36.50) | 36.4 (34.80) | 52.9 (27.17) |
|  | | Median | 34.0 | 25.0 | 55.0 |
|  | | Min, Max | 1, 198 | 1, 152 | 8, 114 |
|  | |  |  |  |  |
| 5 H POSTDOSE | | n | 33 | 33 | 33 |
|  | | Mean (SD) | 31.9 (21.12) | 33.0 (27.60) | 43.9 (24.45) |
|  | | Median | 27.0 | 28.0 | 43.0 |
|  | | Min, Max | 2, 80 | 1, 100 | 8, 103 |
|  | |  |  |  |  |
| 5.5 H POSTDOSE | | n | 33 | 33 | 33 |
|  | | Mean (SD) | 25.5 (17.96) | 30.8 (24.94) | 42.7 (24.03) |
|  | | Median | 21.0 | 26.0 | 42.0 |
|  | | Min, Max | 1, 72 | 1, 98 | 6, 96 |
|  | |  |  |  |  |
| 6 H POSTDOSE | | n | 33 | 33 | 33 |
|  | | Mean (SD) | 19.0 (14.05) | 25.8 (22.46) | 37.9 (24.29) |
|  | | Median | 16.0 | 19.0 | 38.0 |
|  | | Min, Max | 1, 47 | 1, 84 | 5, 110 |
|  |  |  |  |  |  |

***b.) Breath hydrogen concentrations by treatment and timepoint in Booster Omega***

|  |  |  |  |  |  |
| --- | --- | --- | --- | --- | --- |
| Time point | | Statistics | Bi-07 (N=32) | Lactase (N=34) | Placebo (N=34) |
| BASELINE (MEAN) | | N | 32 | 34 | 34 |
|  | | Mean (SD) | 7.7 (5.30) | 7.2 (5.87) | 6.3 (5.53) |
|  | | Median | 6.3 | 5.0 | 4.0 |
|  | | Min, Max | 1, 18 | 1, 18 | 1, 19 |
|  | |  |  |  |  |
| LACTOSE CHALLENGE | | n | 32 | 34 | 34 |
|  | | Mean (SD) | 9.0 (7.18) | 7.5 (5.44) | 6.6 (5.51) |
|  | | Median | 6.5 | 7.0 | 4.0 |
|  | | Min, Max | 2, 28 | 1, 19 | 1, 20 |
|  | |  |  |  |  |
| 0.5 H POSTDOSE | | n | 32 | 34 | 34 |
|  | | Mean (SD) | 9.8 (11.18) | 6.9 (5.45) | 7.4 (5.27) |
|  | | Median | 6.5 | 4.5 | 6.0 |
|  | | Min, Max | 1, 56 | 0, 21 | 1, 21 |
|  | |  |  |  |  |
| 1 H POSTDOSE | | n | 32 | 34 | 34 |
|  | | Mean (SD) | 20.0 (28.65) | 11.4 (14.90) | 20.5 (21.71) |
|  | | Median | 10.0 | 7.5 | 10.5 |
|  | | Min, Max | 1, 142 | 0, 61 | 1, 67 |
|  | |  |  |  |  |
| 1.5 H POSTDOSE | | n | 32 | 34 | 34 |
|  | | Mean (SD) | 31.0 (34.74) | 21.0 (24.16) | 41.4 (49.38) |
|  | | Median | 16.0 | 13.0 | 27.5 |
|  | | Min, Max | 0, 143 | 1, 103 | 1, 230 |
|  | |  |  |  |  |
| 2 H POSTDOSE | | n | 32 | 34 | 34 |
|  | | Mean (SD) | 36.5 (37.73) | 31.3 (37.50) | 55.1 (49.51) |
|  | | Median | 27.5 | 21.5 | 47.0 |
|  | | Min, Max | 0, 129 | 1, 200 | 1, 218 |
|  | |  |  |  |  |
| 2.5 H POSTDOSE | | n | 32 | 34 | 34 |
|  | | Mean (SD) | 37.6 (33.76) | 43.5 (43.12) | 60.0 (41.27) |
|  | | Median | 28.0 | 30.0 | 59.0 |
|  | | Min, Max | 0, 132 | 1, 183 | 1, 169 |
|  |  |  |  |  |  |
|  |  |  |  |  |  |
|  |  |  |  |  |  |
|  |  |  |  |  |  |
|  |  |  |  |  |  |
|  |  |  |  |  |  |

| Time point | | Statistics | Bi-07 (N=32) | Lactase (N=34) | Placebo (N=34) |
| --- | --- | --- | --- | --- | --- |
| 3 H POSTDOSE | | n | 32 | 34 | 34 |
|  | | Mean (SD) | 35.5 (28.37) | 46.3 (39.93) | 63.5 (44.59) |
|  | | Median | 32.0 | 36.0 | 53.0 |
|  | | Min, Max | 0, 90 | 1, 148 | 1, 177 |
|  | |  |  |  |  |
| 3.5 H POSTDOSE | | n | 32 | 34 | 34 |
|  | | Mean (SD) | 33.1 (30.25) | 48.6 (38.59) | 54.5 (39.25) |
|  | | Median | 25.5 | 41.0 | 45.5 |
|  | | Min, Max | 1, 114 | 1, 155 | 3, 191 |
|  | |  |  |  |  |
| 4 H POSTDOSE | | n | 32 | 34 | 34 |
|  | | Mean (SD) | 32.4 (27.52) | 45.8 (38.69) | 60.4 (35.02) |
|  | | Median | 25.0 | 38.0 | 53.0 |
|  | | Min, Max | 1, 109 | 1, 192 | 4, 143 |
|  | |  |  |  |  |
| 4.5 H POSTDOSE | | n | 32 | 34 | 34 |
|  | | Mean (SD) | 33.8 (32.33) | 44.8 (36.39) | 59.8 (44.31) |
|  | | Median | 26.0 | 43.0 | 50.5 |
|  | | Min, Max | 0, 145 | 1, 158 | 6, 234 |
|  | |  |  |  |  |
| 5 H POSTDOSE | | n | 32 | 34 | 34 |
|  | | Mean (SD) | 28.6 (24.41) | 41.6 (25.38) | 49.0 (28.96) |
|  | | Median | 25.0 | 43.0 | 44.0 |
|  | | Min, Max | 0, 97 | 3, 100 | 4, 107 |
|  | |  |  |  |  |
| 5.5 H POSTDOSE | | n | 32 | 34 | 34 |
|  | | Mean (SD) | 32.1 (30.00) | 40.4 (24.96) | 46.9 (24.32) |
|  | | Median | 29.0 | 40.0 | 48.5 |
|  | | Min, Max | 1, 130 | 2, 88 | 3, 100 |
|  | |  |  |  |  |
| 6 H POSTDOSE | | n | 32 | 34 | 34 |
|  | | Mean (SD) | 26.3 (19.74) | 36.4 (26.46) | 41.1 (23.09) |
|  | | Median | 20.5 | 31.0 | 43.0 |
|  | | Min, Max | 1, 79 | 3, 111 | 1, 95 |
|  |  |  |  |  |  |

**Supplementary tables 3: Analysis of C_max_ of breath hydrogen concentration (BHC, ppm)**

***a.) Descriptive statistics of C_max_ of BHC by treatment in Booster Alpha***

| Parameter | Statistics | Bi-07 (N=33) | Lactase (N=33) | Placebo (N=33) |  |
| --- | --- | --- | --- | --- | --- |
| Cmax | n | 33 | 33 | 33 |  |
|  | Mean (SD) | 62.2 (32.98) | 48.3 (35.52) | 75.3 (32.99) |  |
|  | CV(%) | 53.0 | 73.5 | 43.8 |  |
|  | Geometric Mean | 54.4 | 35.0 | 67.2 |  |
|  | Median | 57.0 | 43.0 | 72.0 |  |
|  | Min, Max | 9, 198 | 4, 152 | 11, 159 |  |
|  |  |  |  |  |  |

|  | ***b.) Statistical analysis of C_max_ of BHC in Booster Alpha*** | | | | | | | | | | |
| --- | --- | --- | --- | --- | --- | --- | --- | --- | --- | --- | --- |
|  | | | | | | |  |  |  |  |  |
| Effect | | Num DF | Den DF | F-value | | p-value |  |  |  |  |  |
| Sequence | | 5 | 27 | 3.500 | | 0.0144 |  |  |  |  |  |
| Period | | 1 | 59 | 1.062 | | 0.3069 |  |  |  |  |  |
| Treatment | | 2 | 59 | 14.730 | | <.0001 |  |  |  |  |  |
| Baseline | | 1 | 59 | 3.728 | | 0.0583 |  |  |  |  |  |
| Carryover | | 2 | 59 | 3.165 | | 0.0495 |  |  |  |  |  |
|  | | | | | | |  |  |  |  |  |
|  | | | | | Bi-07 (N=33) | | | Lactase (N=33) | Placebo (N=33) |  |  |
| Geometric LSM (95 % CI) | | | | | 51.2 (40.9, 64.2) | | | 34.6 (27.6, 43.3) | 73.0 (58.3, 91.4) |  |  |
| Comparisons | | | | |  | | |  |  |  |  |
| Ratio of Geometric LSM Vs Placebo, 95 % CI | | | | | 0.701 (0.534, 0.921) | | | 0.474 (0.360, 0.624) |  |  |  |
| p-value | | | | | 0.0115 | | | <.0001 |  |  |  |
| Ratio of Geometric LSM Vs Lactase, 95 % CI | | | | | 1.480 (1.124, 1.949) | | |  |  |  |  |
| p-value | | | | | 0.0061 | | |  |  |  |  |
| **______________________________________________________________________________________** | | | | | | | | | | |  |
|  | | | | | | | | | | |  |

***c.) Descriptive statistics of C_max_ of BHC by treatment in Booster Omega***

| Parameter | Statistics | Bi-07 (N=32) | Lactase (N=34) | Placebo (N=34) |  |
| --- | --- | --- | --- | --- | --- |
| C_max_ (ppm) | n | 32 | 34 | 34 |  |
|  | Mean (SD) | 61.4 (43.14) | 72.9 (50.13) | 93.3 (56.18) |  |
|  | CV (%) | 70.3 | 68.8 | 60.2 |  |
|  | Geometric Mean | 42.7 | 54.4 | 76.9 |  |
|  | Median | 54.5 | 61.5 | 79.5 |  |
|  | Min, Max | 2, 145 | 7, 200 | 11, 234 |  |

***d.) Statistical analysis of C_max_ of BHC in Booster Omega***

|  | | | | | | |  |  |  |
| --- | --- | --- | --- | --- | --- | --- | --- | --- | --- |
| Effect | | Num DF | Den DF | F-value | | p-value |  |  |  |
| Sequence | | 5 | 28 | 2.593 | | 0.0476 |  |  |  |
| Period | | 2 | 61 | 2.835 | | 0.0665 |  |  |  |
| Treatment | | 2 | 61 | 9.561 | | 0.0002 |  |  |  |
| Baseline | | 1 | 61 | 7.253 | | 0.0091 |  |  |  |
|  |  |  |  |  |  |  |  |  |  |
|  |  |  |  |  |  |  |  |  |  |
|  | | | | | Bi-07 (N=32) | | | Lactase (N=34) | Placebo (N=34) |
| Geometric LSM (95% CI) | | | | | 41.5 (31.7, 54.3) | | | 53.9 (41.5, 70.1) | 79.0 (60.7, 102.8) |
| Comparisons | | | | |  | | |  |  |
| Ratio of Geometric LSM Vs Placebo, 95% CI | | | | | 0.525 (0.390, 0.706) | | | 0.682 (0.511, 0.911) |  |
| p-value | | | | | <.0001 | | | 0.0104 |  |
| Ratio of Geometric LSM Vs Lactase, 95% CI | | | | | 0.769 (0.573, 1.033) | | |  |  |
| p-value | | | | | 0.0797 | | |  |  |
| **______________________________________________________________________________________** | | | | | | | | | |

**Supplementary tables 4: Cumulative BHC analysis**

***a.) RM-ANCOVA analysis of BHC in Booster Alpha***

**
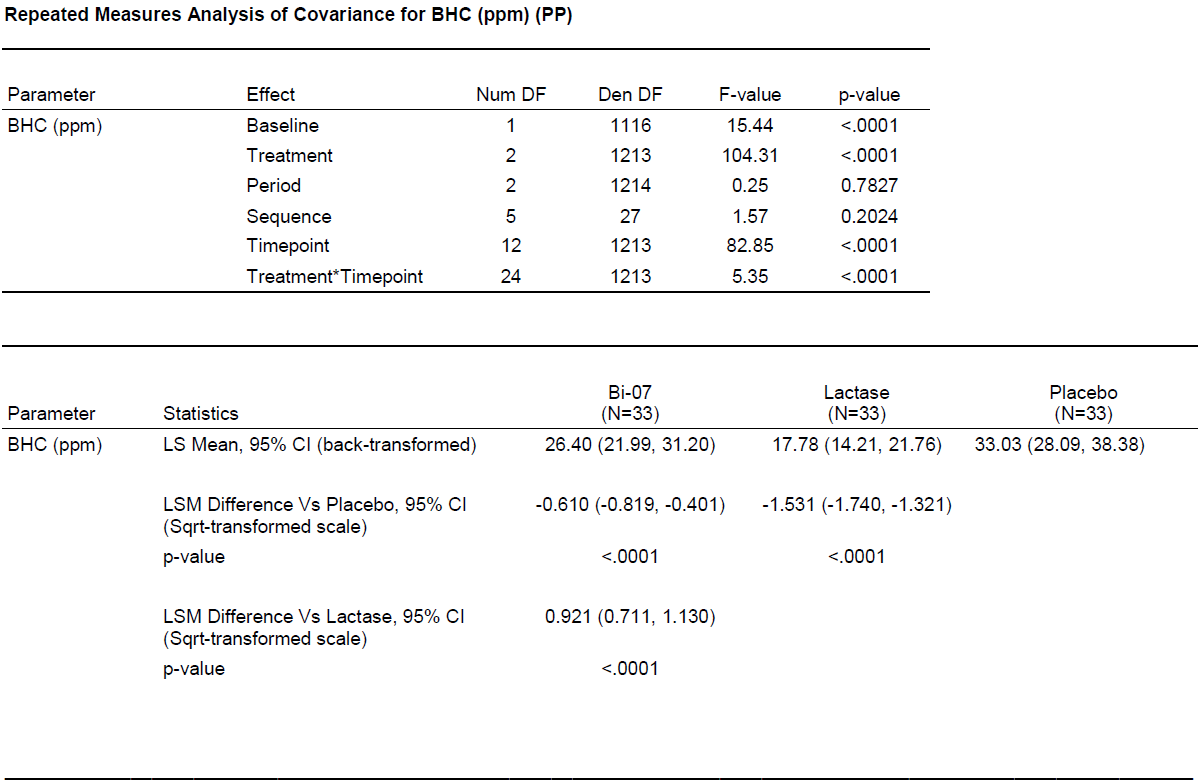
**

***b.) RM-ANCOVA analysis of BHC in Booster Omega***

**
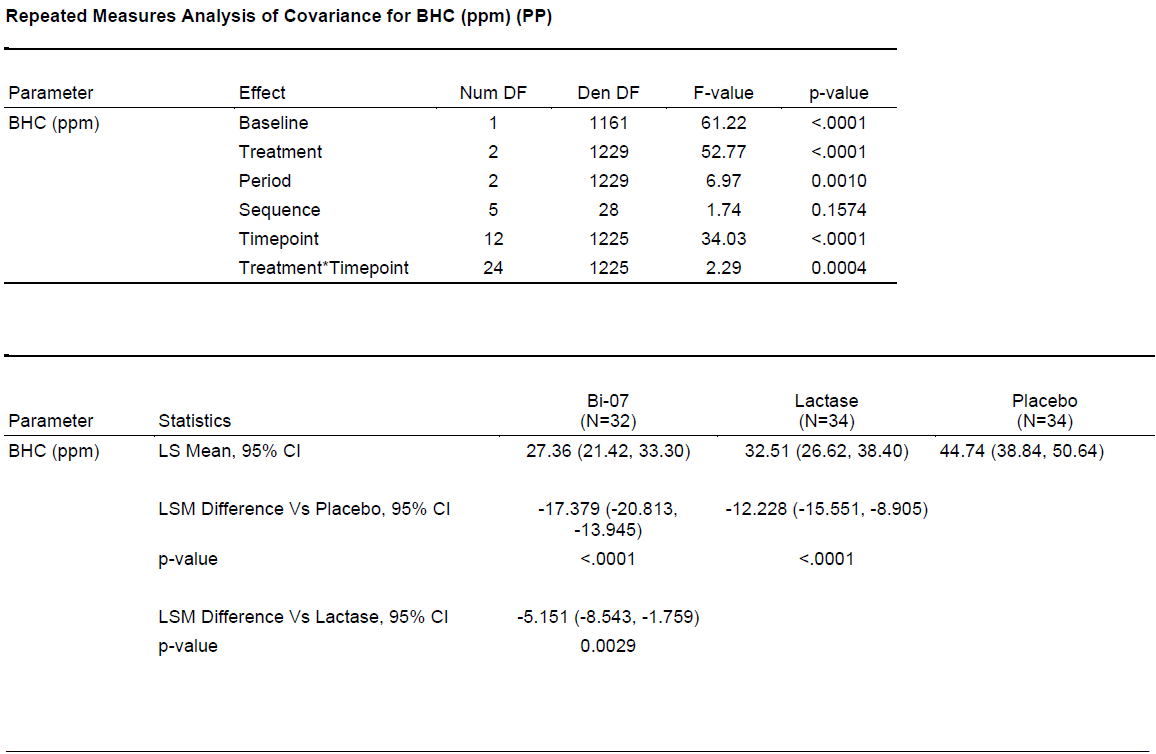
**

**Supplementary tables 5: Descriptive statistics of gastrointestinal symptoms**

1. Descriptive statistics of maximum abdominal pain by treatment in Booster Alpha

|  | | | | | | |
| --- | --- | --- | --- | --- | --- | --- |
|  |  |  |  |  |  |  |
| Parameter | | Findings | Bi-07 (N=33) n (%) | Lactase (N=33) n (%) | Placebo (N=33) n (%) | Screening (N=33) n (%) |
| Maximum abdominal pain | | None | 10 (30.3%) | 16 (48.5%) | 8 (24.2%) | 4 (12.1%) |
|  | | Mild | 13 (39.4%) | 10 (30.3%) | 17 (51.5%) | 15 (45.5%) |
|  | | Moderate | 8 (24.2%) | 7 (21.2%) | 6 (18.2%) | 9 (27.3%) |
|  | | Severe | 2 (6.1%) | 0 (0.0%) | 2 (6.1%) | 5 (15.2%) |
|  | |  |  |  |  |  |
| Change from screening | | -2 | 1 (3.0%) | 4 (12.1%) | 5 (15.2%) |  |
|  | | -1 | 18 (54.5%) | 19 (57.6%) | 9 (27.3%) |  |
|  | | 0 | 9 (27.3%) | 7 (21.2%) | 14 (42.4%) |  |
|  | | 1 | 3 (9.1%) | 3 (9.1%) | 4 (12.1%) |  |
|  | | 2 | 2 (6.1%) | 0 (0.0%) | 1 (3.0%) |  |
|  |  |  |  |  |  |  |
| 0=None, 1=Mild, 2=Moderate, 3=Severe | | | | |  |  |

1. Descriptive statistics of maximum abdominal pain by treatment in Booster Omega

|  |  |  |  |  |  |  |
| --- | --- | --- | --- | --- | --- | --- |
| Parameter | | Findings | Bi-07 (N=32) n (%) | Lactase (N=34) n (%) | Placebo (N=34) n (%) | Screening (N=34) n (%) |
| Maximum abdominal pain | | None | 7 (21.9%) | 10 (29.4%) | 10 (29.4%) | 0 (0.0%) |
|  | | Mild | 13 (40.6%) | 14 (41.2%) | 11 (32.4%) | 13 (38.2%) |
|  | | Moderate | 7 (21.9%) | 9 (26.5%) | 10 (29.4%) | 15 (44.1%) |
|  | | Severe | 5 (15.6%) | 1 (2.9%) | 3 (8.8%) | 6 (17.6%) |
|  | |  |  |  |  |  |
| Change from screening | | -3 | 1 (3.1%) | 2 (5.9%) | 2 (5.9%) |  |
|  | | -2 | 2 (6.3%) | 5 (14.7%) | 5 (14.7%) |  |
|  | | -1 | 13 (40.6%) | 12 (35.3%) | 12 (35.3%) |  |
|  | | 1 | 13 (40.6%) | 13 (38.2%) | 8 (23.5%) |  |
|  | | 2 | 3 (9.4%) | 2 (5.9%) | 7 (20.6%) |  |
|  |  |  |  |  |  |  |
| 0=None, 1=Mild, 2=Moderate, 3=Severe | | | | |  |  |

c) Descriptive statistics of maximum severity of flatulence by treatment in Booster Alpha

|  |  |  |  |  |  |  |  |
| --- | --- | --- | --- | --- | --- | --- | --- |
| Parameter | | Findings | Bi-07 (N=33) n (%) | Lactase (N=33) n (%) | Placebo (N=33) n (%) | | Screening (N=33) n (%) |
| Maximum severity of flatulence | | None | 4 (12.1%) | 9 (27.3%) | 3 (9.1%) | | 1 (3.0%) |
|  | | Mild | 14 (42.4%) | 16 (48.5%) | 15 (45.5%) | | 9 (27.3%) |
|  | | Moderate | 12 (36.4%) | 8 (24.2%) | 11 (33.3%) | | 17 (51.5%) |
|  | | Severe | 3 (9.1%) | 0 (0.0%) | 4 (12.1%) | | 6 (18.2%) |
|  | |  |  |  |  | |  |
| Change from screening | | -3 | 1 (3.0%) | 1 (3.0%) | 0 (0.0%) | |  |
|  | | -2 | 3 (9.1%) | 4 (12.1%) | 3 (9.1%) | |  |
|  | | -1 | 9 (27.3%) | 19 (57.6%) | 11 (33.3%) | |  |
|  | | 0 | 17 (51.5%) | 8 (24.2%) | 16 (48.5%) | |  |
|  | | 1 | 2 (6.1%) | 1 (3.0%) | 1 (3.0%) | |  |
|  | | 2 | 1 (3.0%) | 0 (0.0%) | 2 (6.1%) | |  |
|  |  |  |  |  |  |  |  |
| 0=None, 1=Mild, 2=Moderate, 3=Severe | | | | | |  | |

d) Descriptive statistics of maximum severity of flatulence by treatment in Booster Omega

| p |  |  |  |  |  |  |  |
| --- | --- | --- | --- | --- | --- | --- | --- |
| Parameter | | Findings | Bi-07 (N=32) n (%) | Lactase (N=34) n (%) | Placebo (N=34) n (%) | | Screening (N=34) n (%) |
| Maximum severity of flatulence | | None | 2 (6.3%) | 3 (8.8%) | 6 (17.6%) | | 1 (2.9%) |
|  | | Mild | 17 (53.1%) | 21 (61.8%) | 14 (41.2%) | | 6 (17.6%) |
|  | | Moderate | 9 (28.1%) | 9 (26.5%) | 9 (26.5%) | | 20 (58.8%) |
|  | | Severe | 4 (12.5%) | 1 (2.9%) | 5 (14.7%) | | 7 (20.6%) |
|  | |  |  |  |  | |  |
| Change from screening | | -2 | 2 (6.3%) | 3 (8.8%) | 4 (11.8%) | |  |
|  | | -1 | 15 (46.9%) | 19 (55.9%) | 13 (38.2%) | |  |
|  | | 0 | 13 (40.6%) | 12 (35.3%) | 16 (47.1%) | |  |
|  | | 1 | 2 (6.3%) | 0 (0.0%) | 1 (2.9%) | |  |
|  |  |  |  |  |  |  |  |
| 0=None, 1=Mild, 2=Moderate, 3=Severe | | | | | |  | |

e) Descriptive statistics of maximum severity of bloating by treatment in Booster Alpha

|  |  |  |  |  |  |  |
| --- | --- | --- | --- | --- | --- | --- |
| Parameter | | Findings | Bi-07 (N=33) n (%) | Lactase (N=33) n (%) | Placebo (N=33) n (%) | Screening (N=33) n (%) |
| Maximum severity of bloating | | None | 3 (9.1%) | 5 (15.2%) | 5 (15.2%) | 2 (6.1%) |
|  | | Mild | 12 (36.4%) | 15 (45.5%) | 10 (30.3%) | 13 (39.4%) |
|  | | Moderate | 14 (42.4%) | 9 (27.3%) | 12 (36.4%) | 14 (42.4%) |
|  | | Severe | 4 (12.1%) | 4 (12.1%) | 6 (18.2%) | 4 (12.1%) |
|  | |  |  |  |  |  |
| Change from screening | | -2 | 2 (6.1%) | 3 (9.1%) | 2 (6.1%) |  |
|  | | -1 | 7 (21.2%) | 7 (21.2%) | 7 (21.2%) |  |
|  | | 0 | 14 (42.4%) | 18 (54.5%) | 14 (42.4%) |  |
|  | | 1 | 10 (30.3%) | 5 (15.2%) | 10 (30.3%) |  |
|  |  |  |  |  |  |  |

0=None, 1=Mild, 2=Moderate, 3=Severe

f) Descriptive statistics of maximum severity of bloating by treatment in Booster Omega

|  |  |  |  |  |  |  |
| --- | --- | --- | --- | --- | --- | --- |
| Parameter | | Findings | Bi-07 (N=32) n (%) | Lactase (N=34) n (%) | Placebo (N=34) n (%) | Screening (N=34) n (%) |
| Maximum severity of bloating | | None | 2 (6.3%) | 3 (8.8%) | 7 (20.6%) | 1 (2.9%) |
|  | | Mild | 15 (46.9%) | 20 (58.8%) | 10 (29.4%) | 7 (20.6%) |
|  | | Moderate | 11 (34.4%) | 10 (29.4%) | 12 (35.3%) | 20 (58.8%) |
|  | | Severe | 4 (12.5%) | 1 (2.9%) | 5 (14.7%) | 6 (17.6%) |
|  | |  |  |  |  |  |
| Change from screening | | -2 | 1 (3.1%) | 4 (11.8%) | 6 (17.6%) |  |
|  | | -1 | 15 (46.9%) | 15 (44.1%) | 7 (20.6%) |  |
|  | | 0 | 13 (40.6%) | 14 (41.2%) | 18 (52.9%) |  |
|  | | 1 | 2 (6.3%) | 1 (2.9%) | 3 (8.8%) |  |
|  | | 2 | 1 (3.1%) | 0 (0.0%) | 0 (0.0%) |  |
|  |  |  |  |  |  |  |

0=None, 1=Mild, 2=Moderate, 3=Severe

g) Descriptive statistics of maximum severity of nausea by treatment in Booster Alpha

| Parameter | Findings | Bi-07 (N=33) n (%) | Lactase (N=33) n (%) | Placebo (N=33) n (%) | Screening (N=33) n (%) |  |  |
| --- | --- | --- | --- | --- | --- | --- | --- |
| Maximum severity of nausea | None | 13 (39.4%) | 17 (51.5%) | 13 (39.4%) | 13 (39.4%) |  |  |
|  | Mild | 14 (42.4%) | 13 (39.4%) | 13 (39.4%) | 14 (42.4%) |  |  |
|  | Moderate | 4 (12.1%) | 2 (6.1%) | 6 (18.2%) | 5 (15.2%) |  |  |
|  | Severe | 2 (6.1%) | 1 (3.0%) | 1 (3.0%) | 1 (3.0%) |  |  |
|  |  |  |  |  |  |  |  |
| Change from screening | -3 | 0 (0.0%) | 1 (3.0%) | 1 (3.0%) |  |  |  |
|  | -2 | 1 (3.0%) | 1 (3.0%) | 0 (0.0%) |  |  |  |
|  | -1 | 5 (15.2%) | 7 (21.2%) | 10 (30.3%) |  |  |  |
|  | 0 | 19 (57.6%) | 19 (57.6%) | 10 (30.3%) |  |  |  |
|  | 1 | 8 (24.2%) | 5 (15.2%) | 10 (30.3%) |  |  |  |
|  | 2 | 0 (0.0%) | 0 (0.0%) | 2 (6.1%) |  |  |  |
| 0=None, 1=Mild, 2=Moderate, 3=Severe | | | | | | | |

h) Descriptive statistics of maximum severity of nausea by treatment in Booster Omega

| Parameter | Findings | Bi-07 (N=32) n (%) | Lactase (N=34) n (%) | Placebo (N=34) n (%) | Screening (N=34) n (%) |  |  |
| --- | --- | --- | --- | --- | --- | --- | --- |
| Maximum severity of nausea | None | 7 (21.9%) | 19 (55.9%) | 17 (50.0%) | 12 (35.3%) |  |  |
|  | Mild | 13 (40.6%) | 12 (35.3%) | 12 (35.3%) | 8 (23.5%) |  |  |
|  | Moderate | 10 (31.3%) | 2 (5.9%) | 2 (5.9%) | 13 (38.2%) |  |  |
|  | Severe | 2 (6.3%) | 1 (2.9%) | 3 (8.8%) | 1 (2.9%) |  |  |
|  |  |  |  |  |  |  |  |
| Change from screening | -2 | 2 (6.3%) | 4 (11.8%) | 4 (11.8%) |  |  |  |
|  | -1 | 6 (18.8%) | 13 (38.2%) | 11 (32.4%) |  |  |  |
|  | 0 | 13 (40.6%) | 15 (44.1%) | 12 (35.3%) |  |  |  |
|  | 1 | 9 (28.1%) | 1 (2.9%) | 7 (20.6%) |  |  |  |
|  | 2 | 2 (6.3%) | 1 (2.9%) | 0 (0.0%) |  |  |  |
| 0=None, 1=Mild, 2=Moderate, 3=Severe | | | | | | | |

**Supplementary figures 1: Treatment sequence effect assessment**

1. ***Mean breath hydrogen concentration by sequence (PP) in Booster Alpha***

1. ***Mean breath hydrogen concentration by sequence (PP) in Booster Omega***

B = Bi-07, L = lactase, P = placebo
